# Large deletions in the DNA primase large subunit *PRIM2* are associated with NADP‐malate dehydrogenase activity in a porcine F_2_ cross

**DOI:** 10.1002/age.70077

**Published:** 2026-02-02

**Authors:** Clemens Falker‐Gieske, Iulia Blaj, Ana‐Marija Krizanac, Isabel Kilic, Paula Reich, Jörn Bennewitz, Jens Tetens

**Affiliations:** ^1^ Department of Animal Sciences Georg‐August‐University Göttingen Germany; ^2^ Center for Integrated Breeding Research Georg‐August‐University Göttingen Germany; ^3^ Institute of Animal Breeding and Husbandry Kiel University Kiel Germany; ^4^ Institute of Animal Husbandry and Breeding University of Hohenheim Stuttgart Germany

**Keywords:** genome‐wide association studies, genomics, imputation, pigs, structural variants

## Abstract

Large porcine F_2_ crosses are a valuable resource for discovering QTL and genetic variants for relevant traits. Past studies have been largely limited to SNPs and short insertions and deletions. Structural variants (SVs) are becoming a major area of interest in this respect. Here we present results from a genome‐wide association study with SVs imputed from medium‐density SNP array to the whole genome sequence level that were used to investigate the genetic relationship between important production traits and metabolic enzyme activity in an F_2_ cross based on the breeds Meishan, Piétrain, and European wild boar. Genetic and phenotypic correlations between the two trait classes were high. We were able to pinpoint common genetic loci to a QTL on SSC7, encompassing numerous large intron deletions in the *PRIM2* gene as well as in *HMGCLL1*, *BMP5*, *TRERF1*, *COL21A1*, *LRRC1*, and *UBR2*. The most pronounced genetic associations were observed for the content of NADP‐malate dehydrogenase in the tissue. Hence, we propose that the content and activity of malate dehydrogenase is directly connected to important pig production traits, and we present a comprehensive list of large intronic deletions as promising candidates for causality. The variants were validated in independent pig populations, where the majority of the discovered SVs were present, indicating that they are not only relevant to the breeds investigated here.

## INTRODUCTION

About 2 decades ago, enormous effort was put into the establishment of large resource populations for QTL mapping purposes. Especially in pigs and chickens, huge F_2_ designs were set up and phenotyped in depth for many traits. These designs were mostly analysed using sparse microsatellite marker maps, resulting in large QTL confidence intervals. With the advent of SNP chips and later affordable genome sequencing, some designs were revisited to obtain a higher mapping resolution (Blaj et al., 2018; Stratz et al., 2018), which is most promising in pooled designs (e.g. Falker‐Gieske et al., 2019). However, these studies did not cover the whole depth of genomic variation because they only captured SNPs and short insertions and deletions (InDels) < 50 bp. Structural variants (SVs) have so far been widely neglected due to the obstacles in obtaining high‐confidence variant calls (Ho et al., 2020). As SVs are thought to substantially contribute to complex trait expression (Ho et al., 2020), phenotypic variability (Chen et al., 2024), and gene function (Scott et al., 2021), this variant class is the focus of the study presented here to discover novel and potentially causative QTL. Imputation is a reliable means to improve the marker density in datasets genotyped with low‐density (Daetwyler et al., 2014). Past studies have largely focused on imputing SNPs from array genotype data to whole‐genome sequence (WGS) data. The applicability of imputation between different variant classes, such as SNPs to SVs, was already demonstrated in cattle (Chen et al., 2021), chickens (Falker‐Gieske et al., 2023), and pigs (Falker‐Gieske et al., 2019), with the genotypes from the latter study being the subject of the analysis presented here. Imputation of other variant classes, like microsatellites to SNPs, was performed with high accuracy in horses (Nolte et al., 2022).

Since the precision of genome‐wide association study (GWAS) depends on linkage disequilibrium (LD) block length and the number of individuals studied, single F_2_ populations are only suitable for GWAS to a limited extent. Multiple studies showed that these obstacles can be overcome by pooling closely related populations for GWAS (Bennewitz et al., 2004; Rückert & Bennewitz, 2010; Stratz et al., 2013). The resource populations were often phenotyped for unconventional traits such as ear erectness or hair density, or for physiological and biochemical traits such as enzyme levels. Here, we are particularly analysing muscle enzyme traits in conjunction with fat and muscle traits, such as backfat thickness, in a pooled F_2_‐design (Geldermann et al., 2010). The enzyme traits can be regarded as endophenotypes that are correlated with the target traits, i.e. fat and growth traits, and each explains part of their variation. At the same time, the environmental effect can be supposed to be smaller, as such intermediate phenotypes are conceptually closer to the underlying biological mechanisms. Thus, the heritability can be expected to be higher for the endophenotypes. Furthermore, in a multivariate setting, these traits can be expected to reveal pleiotropic effects as described by Bolormaa et al. (2014) and represent particular metabolic pathways when subjected to principal component analyses (PCAs) (Aschard et al., 2014).

## MATERIALS AND METHODS

### Resource population

The 888 animals used in this study were pooled from three experimental porcine F_2_ crosses, which were established a few decades ago (Geldermann et al., 2003, 2010; Müller et al., 2000). Briefly, three pig F_2_ crosses were generated from three founder breeds: Meishan, Piétrain, and European wild boar, resulting in Meishan × Piétrain, wild boar × Piétrain, and wild boar × Meishan F_1_ animals. For the analyses, only F_2_ animals were used: Meishan × Piétrain (303 individuals), wild boar × Piétrain (283 individuals), and wild boar × Meishan (302 individuals).

### Phenotype data

The traits analysed in this study included several fat and muscle traits: average daily gain (ADG [g/day], post‐weaning), backfat thickness (BFT [mm]), and meat to fat ratio (MFR), and muscle enzyme traits (measured as units/g tissue). These include the biochemical traits soluble protein content of adipose tissue (SPC [mg/g tissue]), glucose‐6‐phosphate dehydrogenase content (G6PD, EC 1.1.1.49), 6‐phosphogluconate dehydrogenase content (6PGD, EC 1.1.1.44), NADP‐isocitrate dehydrogenase content (IDH, EC 1.1.1.42), and NADP‐malate dehydrogenase content (MDH, EC 1.1.1.37) (Geldermann et al., 2003, 2010; Müller et al., 2000). The phenotypes were pre‐corrected for systematic effects (e.g., stable, slaughter month) and the effect of the *RYR1* gene using a general linear model (Blaj et al., 2018).

### Genotype data

Imputed SV and SNP array genotypes (Illumina PorcineSNP60 BeadChip, 61 565 SNPs) were taken from our previous studies (Blaj et al., 2022; Falker‐Gieske et al., 2019). Briefly, 24 founder animals were whole‐genome sequenced at high coverage, 91 F_1_ pigs were sequenced at low coverage, and 2657 F_2_ pigs were SNP genotyped with 60k marker density. In these two studies, animals from a fourth study were included (Boysen et al., 2010). These additional animals were included in the genotype imputation to improve the imputation outcome, but they were not part of the GWAS presented here. SVs were called from the founder animals by combining the results from three different variant callers with SURVIVOR (Blaj et al., 2022). Subsequently, the SVs were combined with the SNPs and short InDels called using the Genome Analysis Toolkit within the scope of our previous study (Falker‐Gieske et al., 2019). This combined dataset served as the reference panel for imputing the F_1_ and F_2_ genotypes to whole‐genome density. SNPs and short InDels were removed from the final genotype set, yielding 6965 SVs for downstream analyses. SV genotypes of 37 control animals of other populations and breeds were called from WGS data published by Groenen et al. (2012). In principle, the same calling strategy was applied, but the union of only two SV callers was used.

### Data analysis

Phenotypic correlations between traits were calculated in R v4.2.1 (R Core Team, 2022). The variance explained by all SNPs (SNP‐based heritability) as well as genomic correlations were estimated using uni‐ and bivariate GREML runs in GCTA v1.92.3 beta3 from 60k SNP array data of F_2_ animals. GWAS was performed on imputed SVs from our previous study (Blaj et al., 2022). For GWAS, a mixed linear model incorporating a “leaving‐one‐chromosome‐out” approach was applied, following the model: y = a + bx + c + g^−^ + e, where y represents the adjusted phenotype, a denotes the overall mean, b is the fixed additive effect of the SV under investigation, c is the fixed effect of the respective crosses, × is the genotype indicator (coded as 0, 1, or 2), and g^−^ corresponds to the random polygenic effect derived from all SNPs excluding those located on the same chromosome as the tested SV. The respective crosses were included in the model as fixed effects. To implement leaving‐one‐chromosome‐out, separate genomic relationship matrices were constructed from F_2_ 60k SNP chip data by systematically omitting one chromosome at a time. Only imputed SVs with a minor allele frequency of at least 1% were included in the model (Blaj et al., 2022; Falker‐Gieske et al., 2019). Variants with *p*‐values below the Bonferroni threshold were considered significant. The single‐trait GWAS results were used to perform a multi‐trait GWAS (mtGWAS) according to Bolormaa et al. (2014). PCAs were conducted in R v4.2.1 for (i) all eight traits (PC_All), (ii) only fat and muscle‐related traits (ADG, BFT, MFR; PC_Fat), and (iii) only muscle enzyme traits (G6PD, 6PGD, IDH, MDH; PC_Enzymes). The first three principal components (PCs) of each setting, visualised in Figure S1, were used as input phenotypes for GWAS, following the same approach as described for single traits above, a similar strategy as applied by (Aschard et al., 2014). PLINK v1.9 (Purcell et al., 2007) was used to perform multidimensional scaling (MDS) analysis, and MDS and Manhattan plots were created with R v4.2.1. Graphical representations of genes were created with SnapGene software v.7.1.1 (www.snapgene.com).

## RESULTS

To investigate if pig carcass traits, especially fat traits, are connected to the activity of metabolic enzymes, we correlated different traits from both categories. Table 1 shows the SNP‐based heritabilities along with the phenotypic and genomic correlations between the traits under investigation. The SNP‐based heritabilities ranged from 0.23 for 6PGD to 0.61 for BFT, with standard errors being sufficiently small. The phenotypic correlations between the enzyme traits were all significant and above 0.5, and they aligned well with the genomic correlations. Hence, we can conclude that there is a high probability that traits under investigation are connected on a genetic and phenotypic level.

**TABLE 1:** Phenotypic and genomic correlations between production and metabolic enzyme traits. SNP‐based heritabilities are on the diagonal (standard errors of the mean in brackets), phenotypic correlations are above, and genomic correlations from bivariate genome‐based restricted maximum likelihood analysis are below the diagonal.

	6PGD	G6PD	IDH	MDH	SPC	MFR	BFT	ADG
6PGD	0.23 (0.06)	0.58 (0.03)	0.53 (0.03)	0.57 (0.03)	−0.32 (0.03)	0.09 (0.03)	0.14 (0.03)	0.06 (0.03)
G6PD	0.56 (0.11)	0.38 (0.06)	0.64 (0.03)	0.64 (0.03)	−0.42 (0.03)	0.10 (0.03)	0.17 (0.03)	0.04 (0.03)
IDH	0.60 (0.10)	0.66 (0.07)	0.46 (0.05)	0.56 (0.03)	−0.25 (0.03)	0.02 (0.03)	0.07 (0.03)	0.02 (0.03)
MDH	0.51 (0.13)	0.54 (0.10)	0.39 (0.11)	0.30 (0.06)	−0.49 (0.03)	0.17 (0.03)	0.30 (0.03)	0.10 (0.03)
SPC	−0.30 (0.15)	−0.53 (0.10)	−0.25 (0.12)	−0.72 (0.09)	0.40 (0.06)	−0.25 (0.03)	−0.36 (0.03)	−0.21 (0.03)
MFR	0.05 (0.15)	0.13 (0.12)	0.01 (0.11)	0.49 (0.11)	−0.39 (0.11)	0.54 (0.05)	0.57 (0.03)	−0.05 (0.03)
BFT	0.12 (0.13)	0.19 (0.11)	0.02 (0.10)	0.53 (0.10)	−0.51 (0.09)	0.60 (0.07)	0.61 (0.05)	−0.02 (0.03)
ADG	−0.08 (0.16)	−0.23 (0.13)	−0.02 (0.12)	−0.31 (0.14)	0.19 (0.13)	−0.29 (0.11)	−0.36 (0.10)	0.42 (0.06)

Abbreviations: 6PGD, 6‐phosphogluconate dehydrogenase; G6PD, glucose‐6‐phosphate dehydrogenase; IDH, NADP‐isocitrate dehydrogenase; MDH, NADP‐malate dehydrogenase; SPC. soluble protein content of adipose tissue; MFR, meat to fat ratio; BFT, back fat thickness; ADG, average daily gain.

**FIGURE 1:**
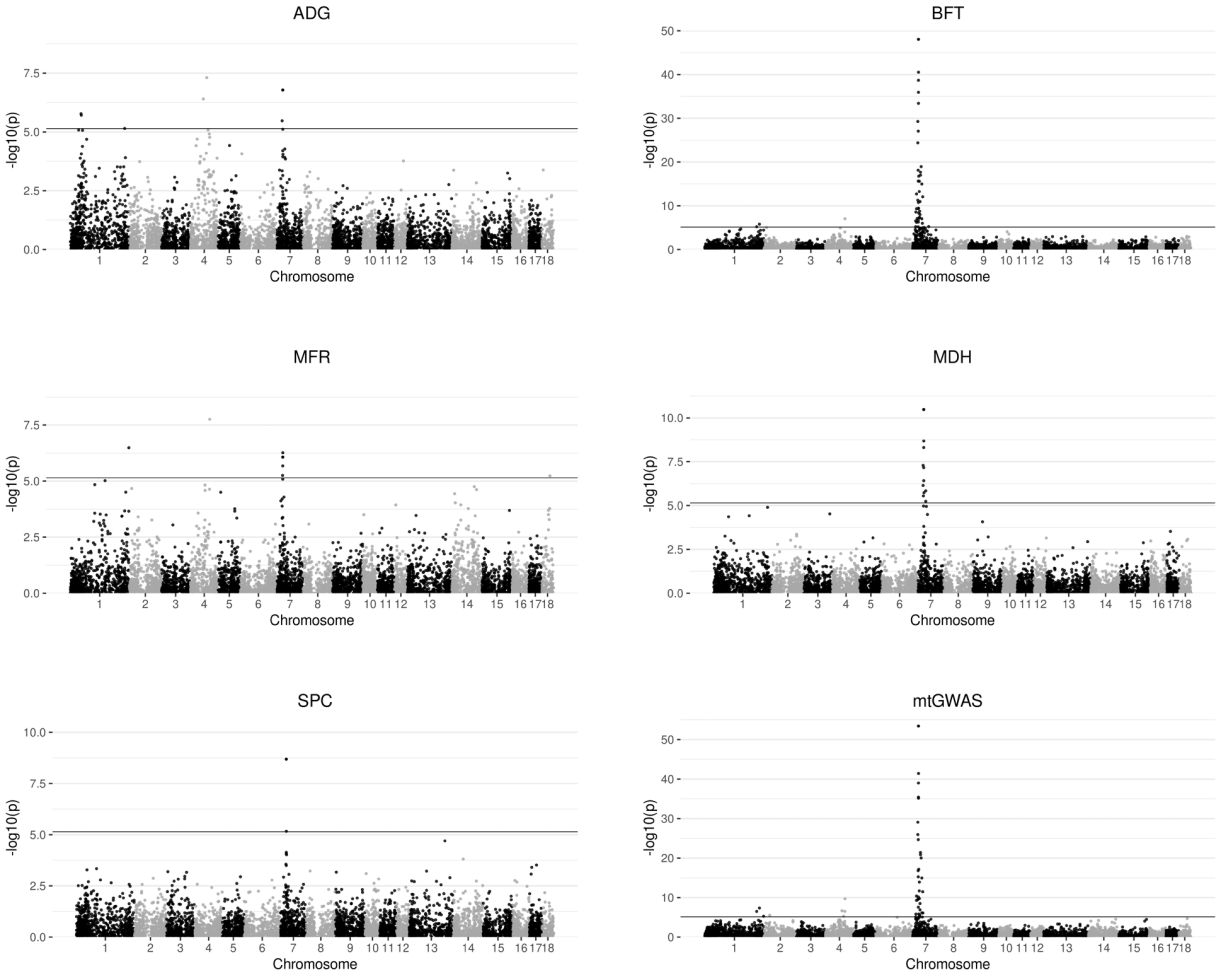
Manhattan plots of genome‐wide association studies with structural variants imputed from medium SNP array density to whole‐genome level for the traits average daily gain (ADG), back fat thickness (BFT), meat to fat ratio (MFR), NADP‐malate dehydrogenase content (MDH), and soluble protein content of adipose tissue (SPC). The multi‐trait GWAS (mtGWAS) was performed on all traits under investigation in this study. The line indicates the Bonferroni significance threshold.

**FIGURE 2:**
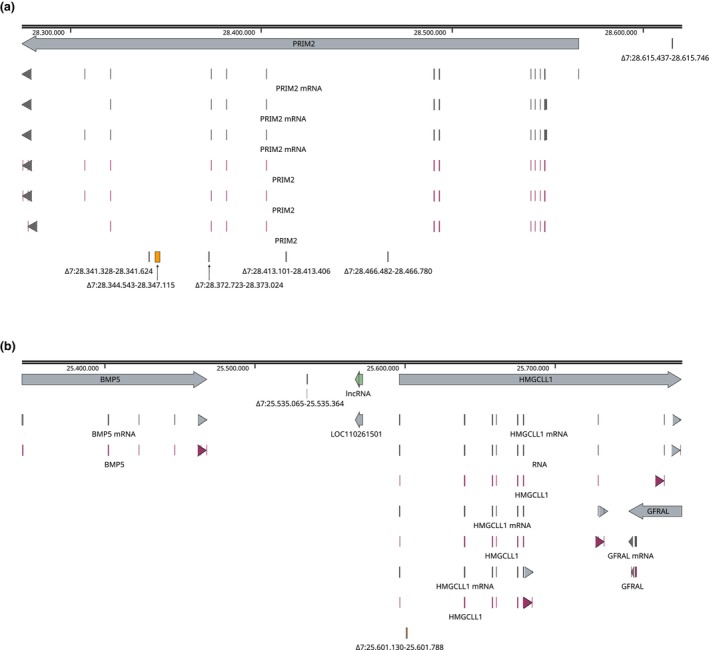
Graphical representation of deletions in PRIM2 (a) and BMP5/HMGCLL1 (b), which were associated (*p*‐value <1 × 10^−6^) with the trait NADP‐malate dehydrogenase content. Grey arrow boxes indicate mRNAs, and violet arrow boxes indicate exons of transcript variants.

Performing GWAS, significant associations for most of the traits were discovered on SSC7 (Figure 1). As ADG, BFT, and MFR were already investigated in detail in our previous study (Blaj et al., 2022), the focus of the present study will be on the biochemical traits. No significant GWAS signals were detected for the traits 6PGD, G6PD, and IDH (Figure [Link], [Link]). Among the significantly associated SVs for the trait MDH, we exclusively discovered deletions in the size range of 83–2572 bp, which were mostly located in introns (Table 2). The dosage R‐squared values of these variants, which are a measure of imputation accuracy, range from 0.334 to 0.941 (average 0.612). The three top variants were also associated with the trait SPC. Five intronic deletions and one 5′‐untranslated region deletion were located in the *PRIM2* gene (Figure 2a). Furthermore, one intronic deletion was discovered in the *HMGCLL1* gene, and one intergenic deletion between *BMP5* and *HMGCLL1* (Figure 2b). The top variant found in the mtGWAS was identical to the one found for MDH, and the two top variants from mtGWAS were commonly above the Bonferroni threshold in the traits ADG, BFT, MFR, MDH, and SPC (Figure S3). Both are intronic deletions in the *PRIM2* gene with fairly high allele frequencies between 0.69 and 0.73.

**TABLE 2:** Large deletions on SSC7 that were significantly associated with NADP‐malate dehydrogenase content (MDH) in the back fat layer of pigs.

Position (bp)	*p*‐value	Size (bp)	Gene	Consequence	AF (founders)	AF (controls)	DR^2^
28 372 723	3.31 × 10^−11^	301	*PRIM2*	Intron	0.73	0.20	0.941
28 413 101	3.32 × 10^−11^	305	*PRIM2*	Intron	0.69	0.25	0.785
28 615 437	2.10 × 10^−9^	309	*PRIM2*	5′‐UTR	0.67	/	0.618
28 344 543	4.96 × 10^−9^	2572	*PRIM2*	Intron	0.59	/	0.801
25 601 130	5.20 × 10^−8^	658	*HMGCLL1*	Intron	0.64	/	0.407
28 341 328	6.95 × 10^−8^	296	*PRIM2*	Intron	0.57	0.11	0.732
28 466 482	3.87 × 10^−7^	298	*PRIM2*	Intron	0.58	0.07	0.651
25 535 065	7.17 × 10^−7^	299	*HMGCLL1‐BMP5*	Intergenic	0.62	0.13	0.334
37 641 459	1.45 × 10^−6^	124	*TRERF1*	Intron	0.93	0.45	0.78
29 528 935	1.85 × 10^−6^	83	*COL21A1*	Intron	0.96	0.34	0.504
26 905 670	2.83 × 10^−6^	283	*LRRC1*	Intron	0.61	0.16	0.353
37 760 958	5.81 × 10^−6^	281	*UBR2*	Intron	0.85	0.41	0.433

Abbreviations: AF, allele frequency; DR^2^, dosage R‐squared; UTR, untranslated region.

In accordance with the results from single‐trait GWAS, significant association signals on SSC7 were also detected in the PC‐based GWAS for the first three PCs obtained from all traits (Figure S4). The top seven variants for the first PC were identical to those detected for MDH. A significant association signal on SSC7 was further identified for PC1 and PC3 obtained from the fat traits, and for PC3 derived from the enzyme traits (Figure S4). The proportion of variance explained by single PCs is stated in Table S1. Summary statistics of significant signals for all traits are summarised in Appendix S1 and QQ‐plots as well as λ‐values in Appendix S2.

To validate the practical relevance of the SVs significantly associated with MDH, we tested their segregation in other pig populations. For this, we utilised WGS data from 37 pigs of the breeds Duroc, Hampshire, Jiangquhai, Landrace, Large White, Meishan, Piétrain, and Xiang, which were generated by Groenen et al. (2012). Nine out of 12 SVs, including the two top SVs, were present in these animals, but at lower allele frequencies (Table 2). Furthermore, most of the SVs were only detectable when using the union of two SV callers, which is also explainable by the lower allele frequencies. However, this was to be expected due to the different breed composition of the control population. To validate that the breeds from the experimental and control populations are comparable, MDS analysis was performed. As expected, the breeds Piétrain, Meishan, Landrace, and Large White clustered in the MDS analysis (Figure S5).

## DISCUSSION

This study aimed to test whether production traits in pigs are connected to the content of metabolic enzymes in muscle tissue. GWAS were conducted on three pooled porcine F_2_ populations. Single F_2_ populations are not suitable for GWAS because of their limited genetic diversity, strong LD, non‐random mating and family structure, as well as small effective population size. Therefore, the pooling of multiple crosses was demonstrated to be beneficial in GWAS settings (Bennewitz et al., 2004; Rückert & Bennewitz, 2010; Stratz et al., 2013). Genomic and phenotypic correlations based on SNP array data validate that the fat and biochemical traits under investigation are indeed connected, which is confirmed by the results from different GWAS approaches. Low correlations between ADG and the enzyme traits are not surprising, since ADG is not a direct measure of fat metabolism. Here, we utilised imputed SV genotypes from our previous study (Blaj et al., 2022) for single‐trait GWAS in conjunction with mtGWAS and PCA‐based GWAS. Imputation of low‐density marker panels, like SNP arrays, to higher‐density variants was shown to be effective in numerous studies (reviewed here (Marchini & Howie, 2010)) and also applies to SVs (Blaj et al., 2022; Chen et al., 2021; Falker‐Gieske et al., 2023). We achieved high imputation accuracies, especially among the variants with the lowest *p*‐values, which underscores the reliability of the presented results. With our GWAS strategy, we mostly detected associations with intronic deletions in several genes. The only significant association signal among the biochemical traits was found for the trait MDH. Malate dehydrogenase is involved in the metabolism of pyruvate and carbon fixation and plays an essential role in the tricarboxylic acid (TCA) cycle (Broeks et al., 2019). The TCA cycle is a crucial metabolic pathway that carries out the vital role of oxidising nutrients to sustain the energy needs of cells (reviewed here (Arnold & Finley, 2023)).

We pinpointed the connection between production traits and MDH to two intronic deletions in the *PRIM2* gene, which were commonly significant among these traits in single‐trait GWAS and showed significant associations also in mtGWAS and five out of eight PCA‐based GWAS for all traits (PC1, PC2, PC1_fat, PC3_fat, and PC3_enzymes). Based on the assumption that a low number of top PCs explain the largest part of the complete trait variance, PCA‐based GWAS offers sufficient power to detect true pleiotropic effects (Avery et al., 2011; Zhang et al., 2018). These common associations discovered in the different GWAS approaches, together with the moderate genomic correlations between MDH and fat traits, suggest pleiotropic effects of the commonly detected variants and indicate the existence of metabolic pathways with an influence on both the enzyme and fat traits. Furthermore, half of the SVs associated with MDH were located in the *PRIM2* gene, which encodes the DNA primase large subunit, one of four subunits of the DNA polymerase α complex and is essential for the initiation of DNA synthesis (Schneider et al., 1998). DNA replication is the most important step in the cell cycle, and is crucial for the continuity and stability of the genomes of daughter cells. During this process, *PRIM2* is activated by proliferating‐cell‐nuclear‐antigen (PCNA) (Wang, Tang, et al., 2022). The authors found that *PRIM2* promotes DNA replication and mismatch repair and activates the cell cycle. Its knockdown decreases cell viability and enhances cell death, respectively (Mu et al., 2021). Kaewsutthi et al. suggested *PRIM2* to be associated with human obesity (Kaewsutthi et al., 2016). In livestock, *PRIM2* was associated with body weight and trait changes in pigs (Borowska et al., 2017; Wang et al., 2014) as well as with growth in cattle (An et al., 2020). This body of evidence, combined with the findings presented here, makes the *PRIM2* gene a promising breeding target for improving porcine production traits. Experimental validation of the discovered deletions is warranted to identify true causative variants.

The remaining discussion focuses on the SVs significantly associated with MDH (Table 2) as the fat and growth traits were already subjects of earlier studies (Blaj et al., 2022; Falker‐Gieske et al., 2019). A large intronic deletion of 658 bp in the *HMGCLL1* gene was significantly associated with MHD as well. Its gene product, the 3‐hydroxy‐3‐methylglutaryl‐CoA lyase‐like protein, is a mitochondrial enzyme associated with ketogenesis through the synthesis of acetoacetate and acetyl‐CoA. It might be an active, extramitochondrial HMG‐CoA lyase subjected to post‐translational myristoylation (Montgomery et al., 2012). Hence, an increase in the coenzyme acetyl‐CoA production due to a gain of function in the *HMGCLL1* gene may lead to an increase of malate dehydrogenase activity, which converts malate to oxaloacetate in the citric acid cycle. It has been hypothesised that the cell determines its metabolic state depending on acetyl‐CoA levels (Shi & Tu, 2015). Several studies have already identified *HMGCLL1* to be associated with meat and/or carcass traits in pigs (Falker‐Gieske et al., 2019; Pena et al., 2019) and cattle (Li et al., 2022). This is in line with the results of Montgomery et al. (2012), who suggest plausible physiological roles of human *HMGCLL1* in either lipid biosynthesis or energy metabolism (Montgomery et al., 2012).

An intergenic variant between *HMGCLL1* and *BMP5* showed association with MDH as well. The bone morphogenetic protein 5 belongs to a group of signalling factors that play multifunctional roles during embryonic skeletal development (Bailón‐Plaza et al., 1999). They are involved in a variety of cellular processes, such as proliferation, differentiation, and apoptosis (Hogan, 1996). Different studies implicated *BMP5* in growth, body weight and carcass traits in different livestock species (Chen et al., 2020; Daza et al., 2021; Pérez‐Montarelo et al., 2014; Shao et al., 2011). In pigs, it is located within a major QTL region affecting carcass fat content, and an SNP within *BMP5* has been associated with fatness (Shao et al., 2011). Furthermore, miRNAs influence the translational inhibition of *BMP5*, as its downregulation led to altered fat deposition in pigs (Daza et al., 2021; Shao et al., 2011).

An intronic deletion detected in the *TRERF1* gene showed the highest allele frequency (0.45) among the control group of animals. This implies that this variant is not only relevant to European breeds. *TRERF1* encodes the transcriptional regulating factor for CYP11A1 and has been reported to activate CYP11A1 (Hao et al., 2011), a mitochondrial enzyme that catalyses the cleavage of the side chain of cholesterol to produce pregnenolone, the first synthesis step of all steroid hormones. Electrons for these reactions are provided by NADPH (Gizard et al., 2001; Slominski et al., 2015), indicating a possible link to the TCA cycle.

Another statistically significant intronic deletion with a high allele frequency (0.34) in the control group was located in the *COL21A1* gene, which encodes the collagen type XXI α 1 chain. This variant is in close proximity to already known QTL for fatness traits mapped within *COL21A1* (Huang et al., 2011; Wang, Wang, et al., 2022). *COL21A1* is part of the family of fibril‐associated collagens with interrupted triple helices (FACIT), which are expressed in various tissues (Fitzgerald & Bateman, 2001). When connected to collagen I, collagen XXI plays a significant role in organising interstitial collagen fibrils (Chou & Li, 2002). Furthermore, Chou & Li demonstrated that *COL21A1* is expressed by smooth muscle cells in the artery wall, suggesting that collagen XXI may contribute to the extracellular matrix assembly of the vascular network during blood vessel formation. Wang et al. described two SNPs (SSC7:29503670 and rs1112937671 (SSC7:29486003)) located within an intronic region of the *COL21A1* gene, which were associated with average backfat thickness (Wang, Wang, et al., 2022). They assumed that *COL21A1* might play an important role in the porcine backfat deposition by affecting remodelling and should be considered a strong candidate gene for porcine backfat traits.

Additional candidate genes for MDH affected by intronic deletions were *LRRC1*, which is involved in early‐stage adipocytic differentiation (Wang et al., 2023), and *UBR2*, which encodes a protein that binds leucine and is a negative regulator of the leucine‐mTOR signalling pathway, thereby controlling cell growth (Kume et al., 2010).

## CONCLUSION

The study presented here clearly demonstrates the advantage of revisiting deeply phenotyped QTL‐mapping populations established in the past with recent genomic and statistical methods and approaches. In summary, all the genes harbouring SVs, which were significantly associated with MDH and other traits investigated here, fit the phenotypes under investigation: production traits and traits connected to metabolic activity. Given the complex genetic architecture of these traits, it is expected that multiple associated variants affecting different genes will be found. This study is one step further towards the identification of true causative variants for these traits, as SVs largely contribute to phenotypic variation (Perry et al., 2007; Spielmann et al., 2018; Stankiewicz & Lupski, 2010). It is to be expected that most of the SNPs, which were the main focus of most published mapping studies, are only in LD with the true causative variants. In future studies, we will validate the variants discovered here by conducting CRISPR/Cas9 mediated deletions in porcine cell lines. These experiments will deliver final proof of the causative nature of the SVs to make them desirable breeding targets.

## AUTHOR CONTRIBUTIONS

C.F.G. performed formal data analysis and wrote the manuscript; I.B. conceptualised the study; A.‐M.K. performed multidimensional scaling analysis and plotting; I.K. wrote the manuscript; P.R. performed principal component analyses and contributed to the manuscript; J.B. contributed to the manuscript and data analysis; J.T. supervised the study and developed data analysis strategies.

## FUNDING INFORMATION

The study was funded by the German Research Foundation (Deutsche Forschungsgemeinschaft, DFG). The funding body did not contribute to the design of the study or collection, analysis, and interpretation of data, and writing of the manuscript.

## CONFLICT OF INTEREST STATEMENT

The authors declare to have no competing interests of any kind.

## Supporting information


Appendix S1:

Appendix S2:

Fig. S1:

Fig. S2:

Fig. S3:

Fig. S4:

Fig. S5:

Table S1:

## Data Availability

All the data utilised in this study have been made publicly available earlier and can be found here https://doi.org/10.25387/g3.8287847. The whole genome sequencing data is accessible via BioProject ID PRJNA553106.
