# Supplementary material for: Large deletions in the DNA primase large subunit PRIM2 are associated with NADP‐malate dehydrogenase activity in a porcine F2 cross
Source: Anim Genet. 2026 Feb 2;57(1):e70077. doi: 10.1002/age.70077 (PMC12864183; doi:10.1002/age.70077)
Supplement: Supplementary file 2 — Appendix S2: [file AGE-57-0-s002.pdf]

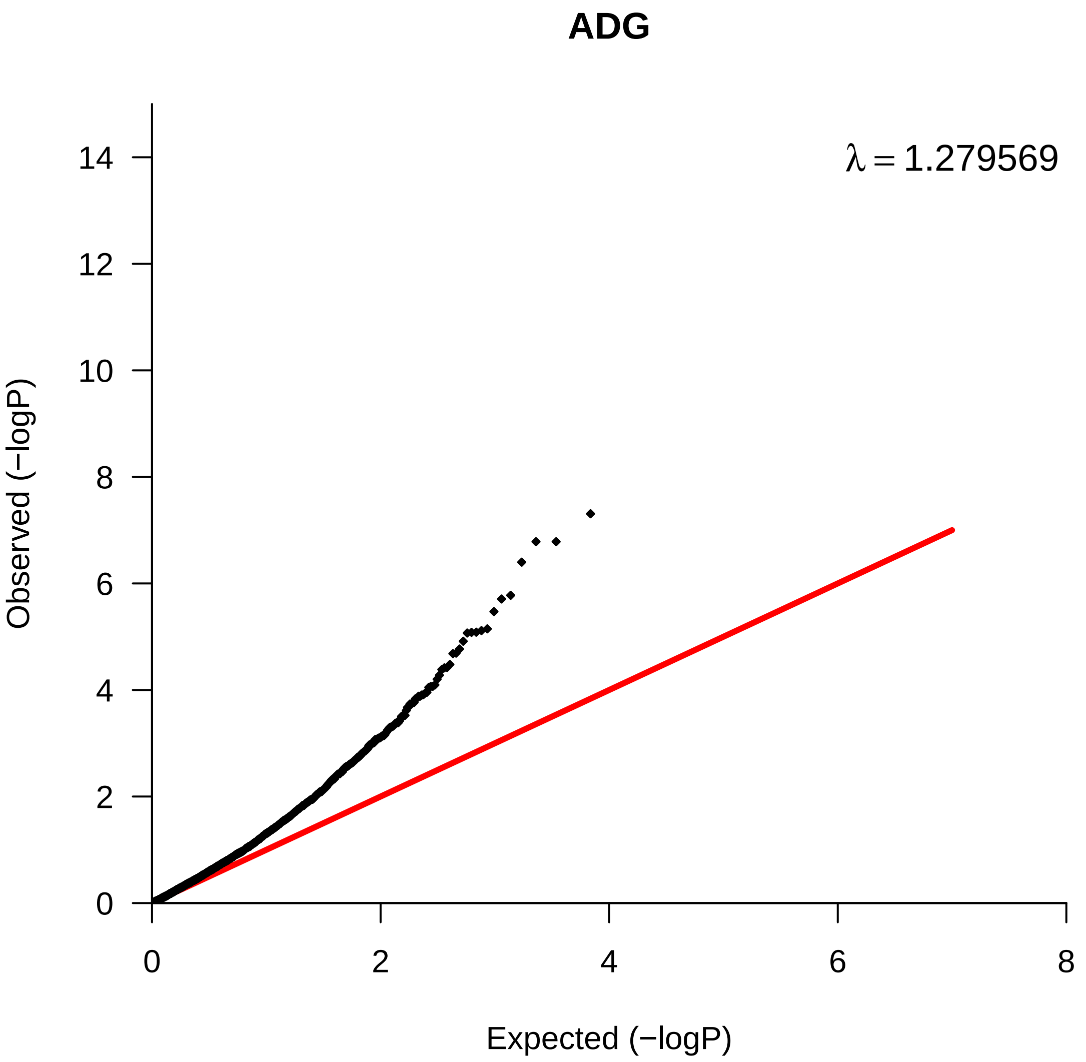
ADG
λ = 1.279569
Observed (−logP)
Expected (−logP)
0
2
4
6
8
10
12
14
0
2
4
6
8

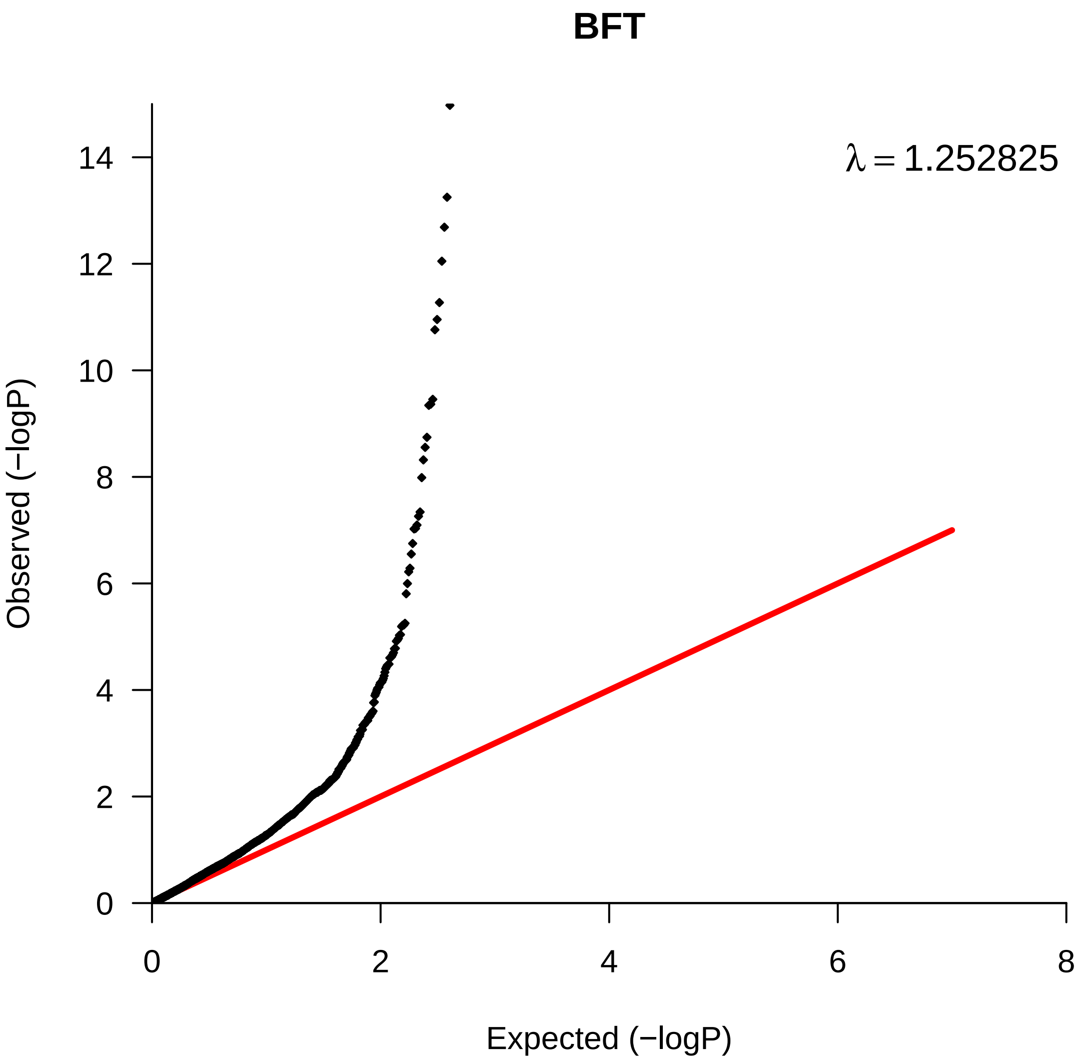

BFT
λ = 1.252825
Observed (−logP)
Expected (−logP)
0
2
4
6
8
10
12
14
0
2
4
6
8

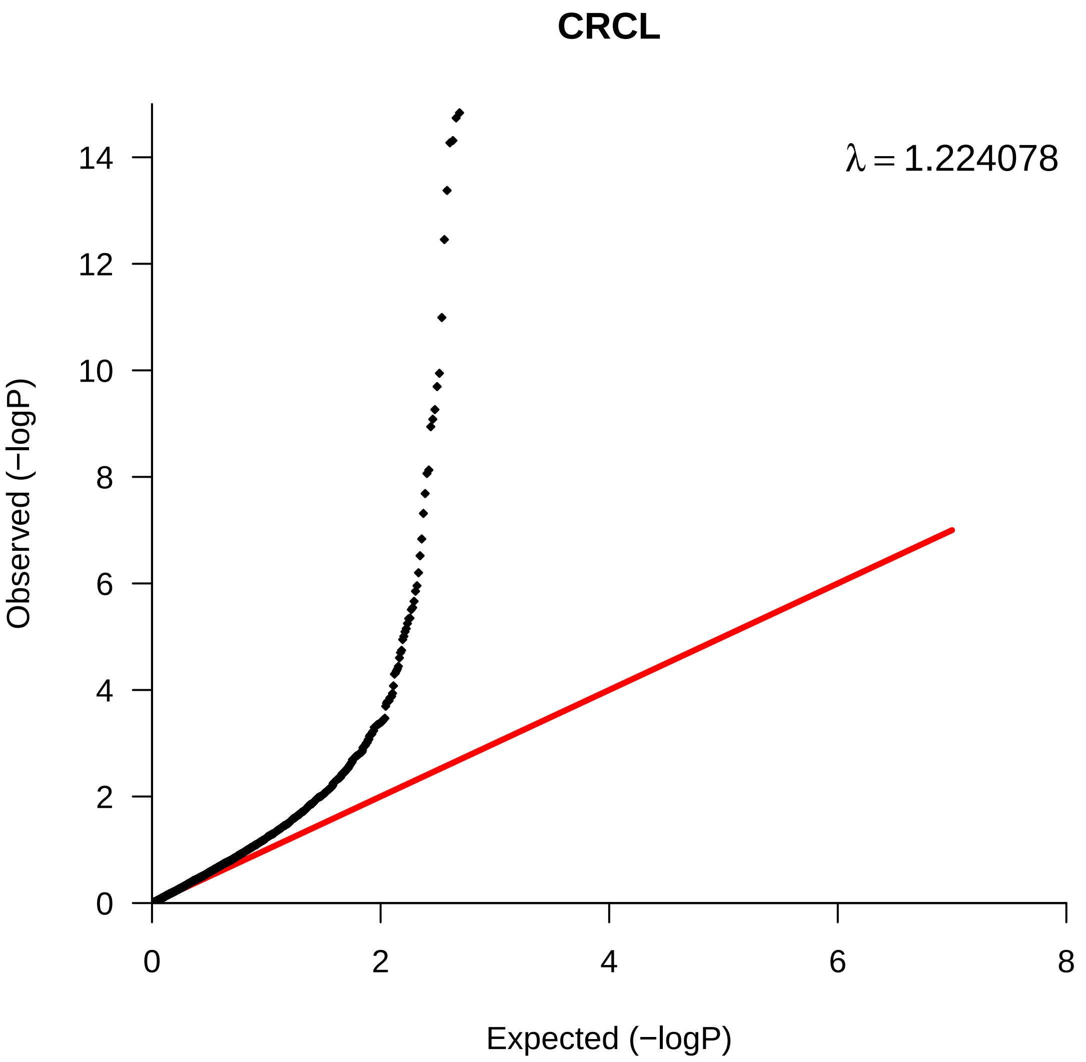
CRCL
λ = 1.224078
Observed (−logP)
Expected (−logP)
0
2
4
6
8
10
12
14
0
2
4
6
8

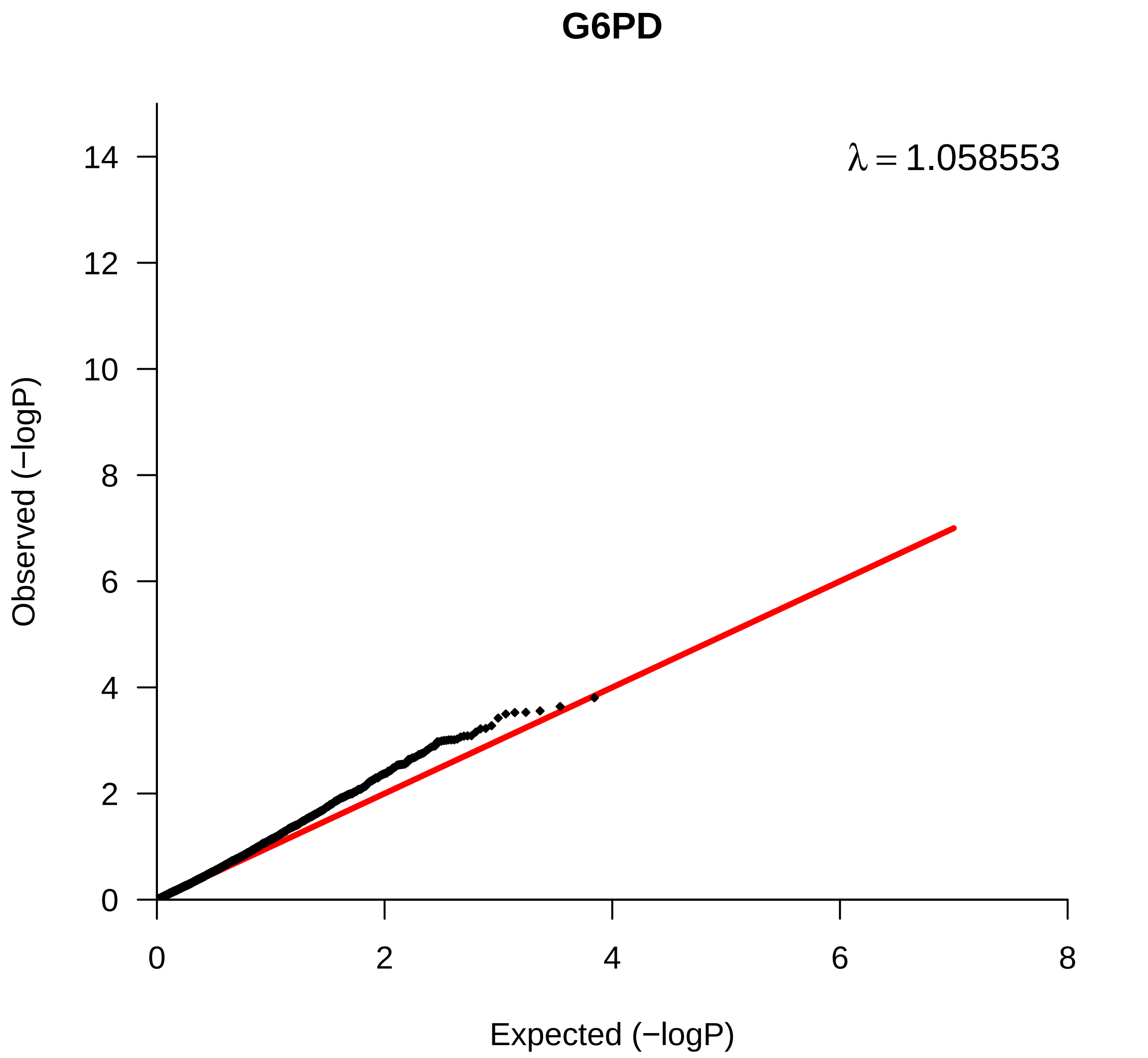
G6PD
λ = 1.058553
Observed (−logP)
Expected (−logP)
0
2
4
6
8
10
12
14
0
2
4
6
8

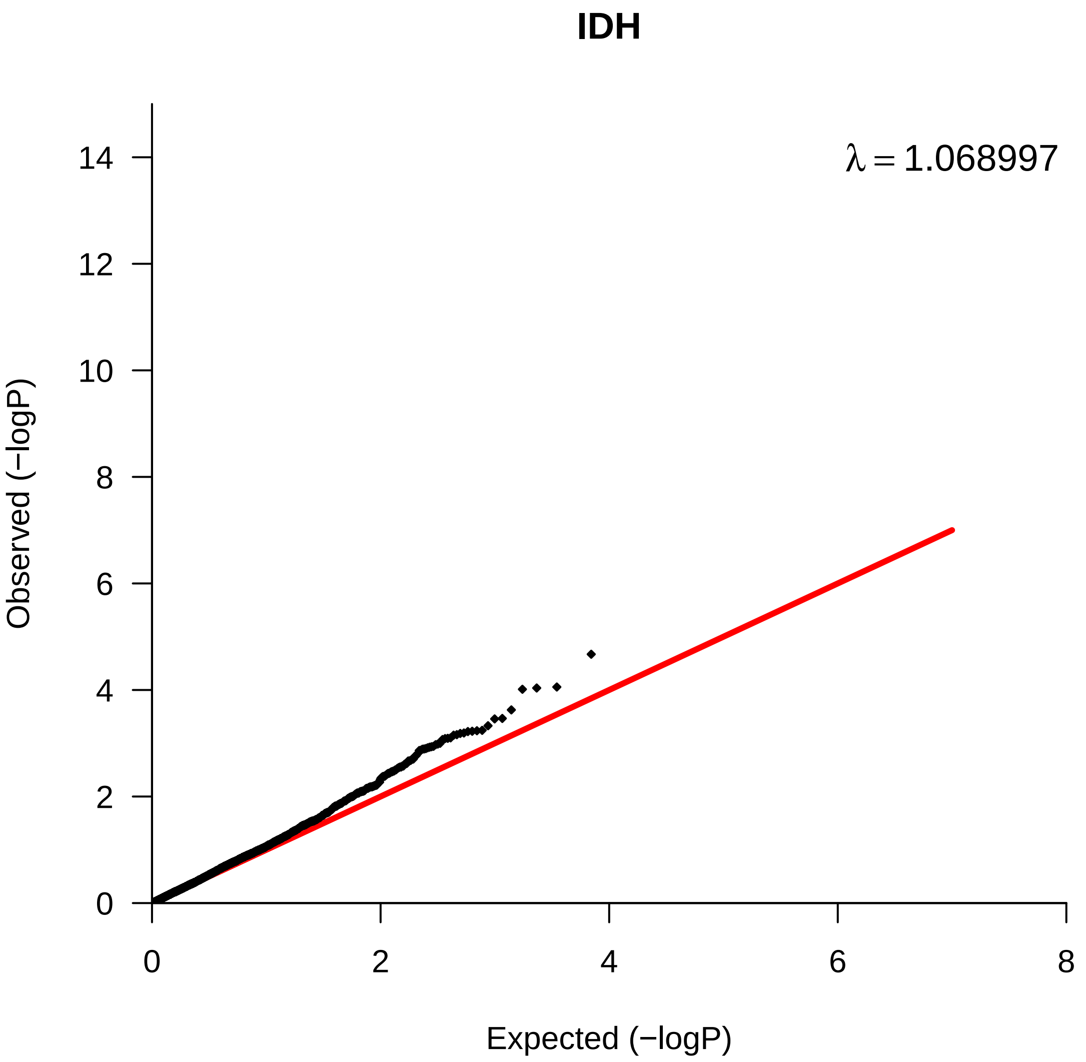
IDH
λ = 1.068997
Observed (−logP)
Expected (−logP)
0
2
4
6
8
10
12
14
0
2
4
6
8

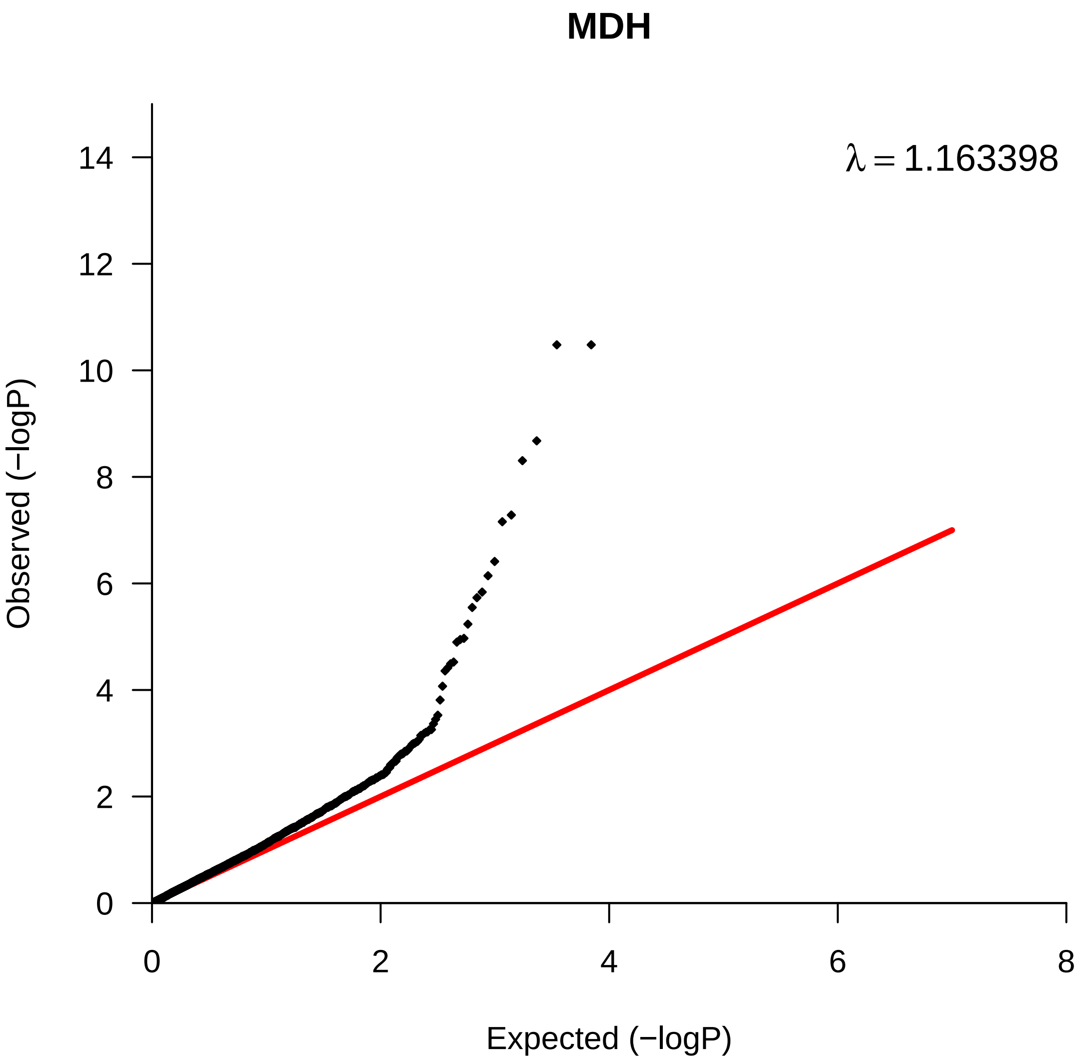

MDH
λ = 1.163398
Observed (−logP)
Expected (−logP)
0
2
4
6
8
10
12
14
0
2
4
6
8

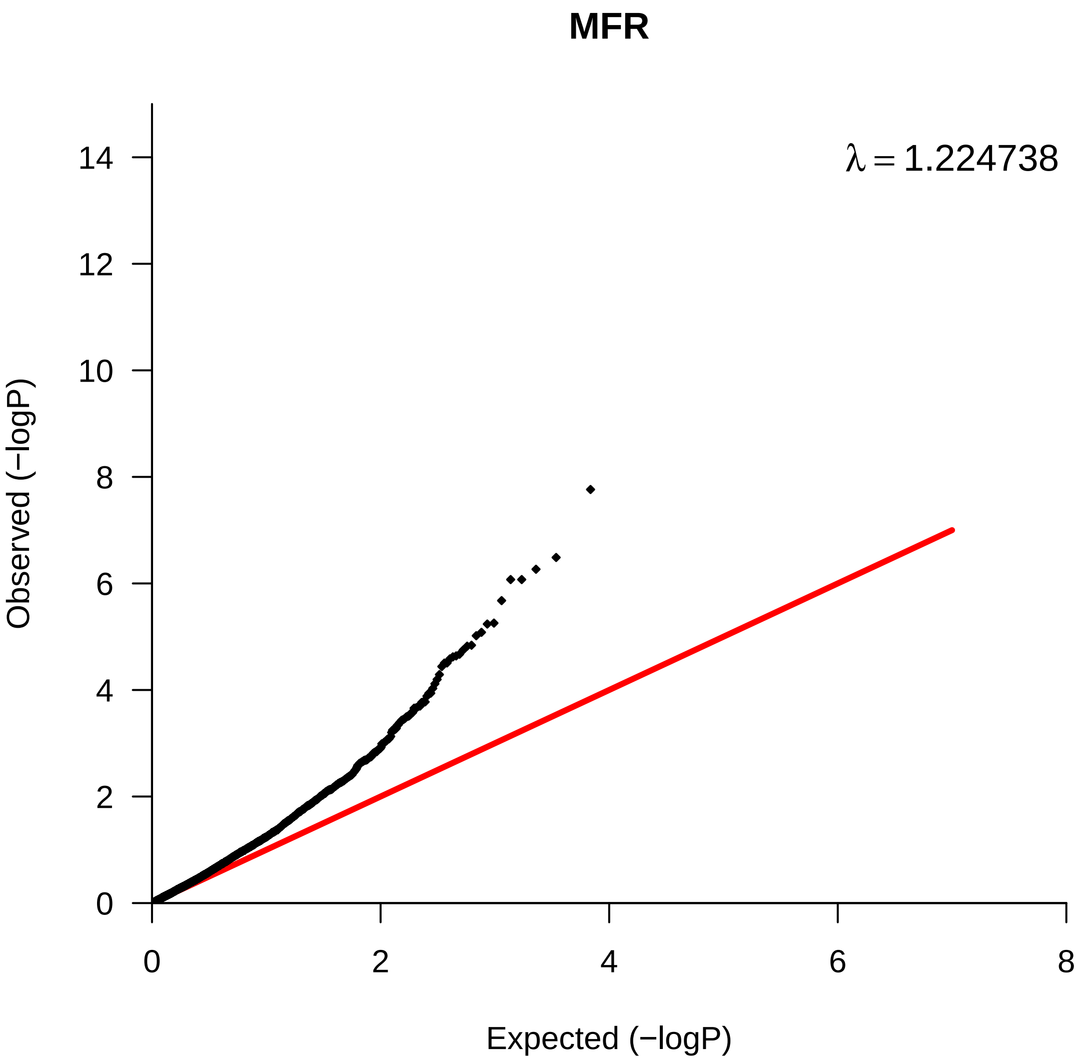

MFR
λ = 1.224738
Observed (−logP)
Expected (−logP)
0
2
4
6
8
10
12
14
0
2
4
6
8

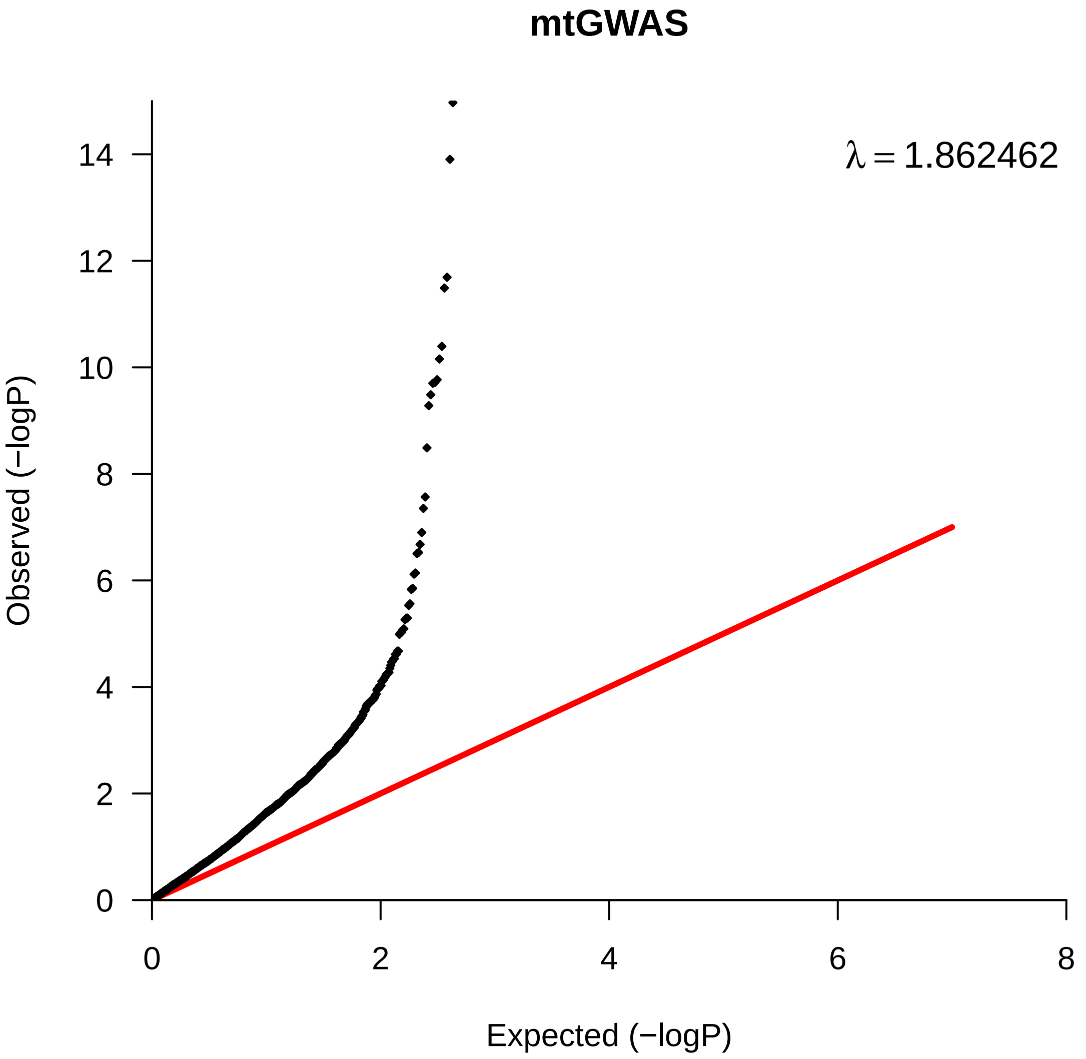
mtGWAS
λ = 1.862462
Observed (−logP)
Expected (−logP)
0
2
4
6
8
10
12
14
0
2
4
6
8

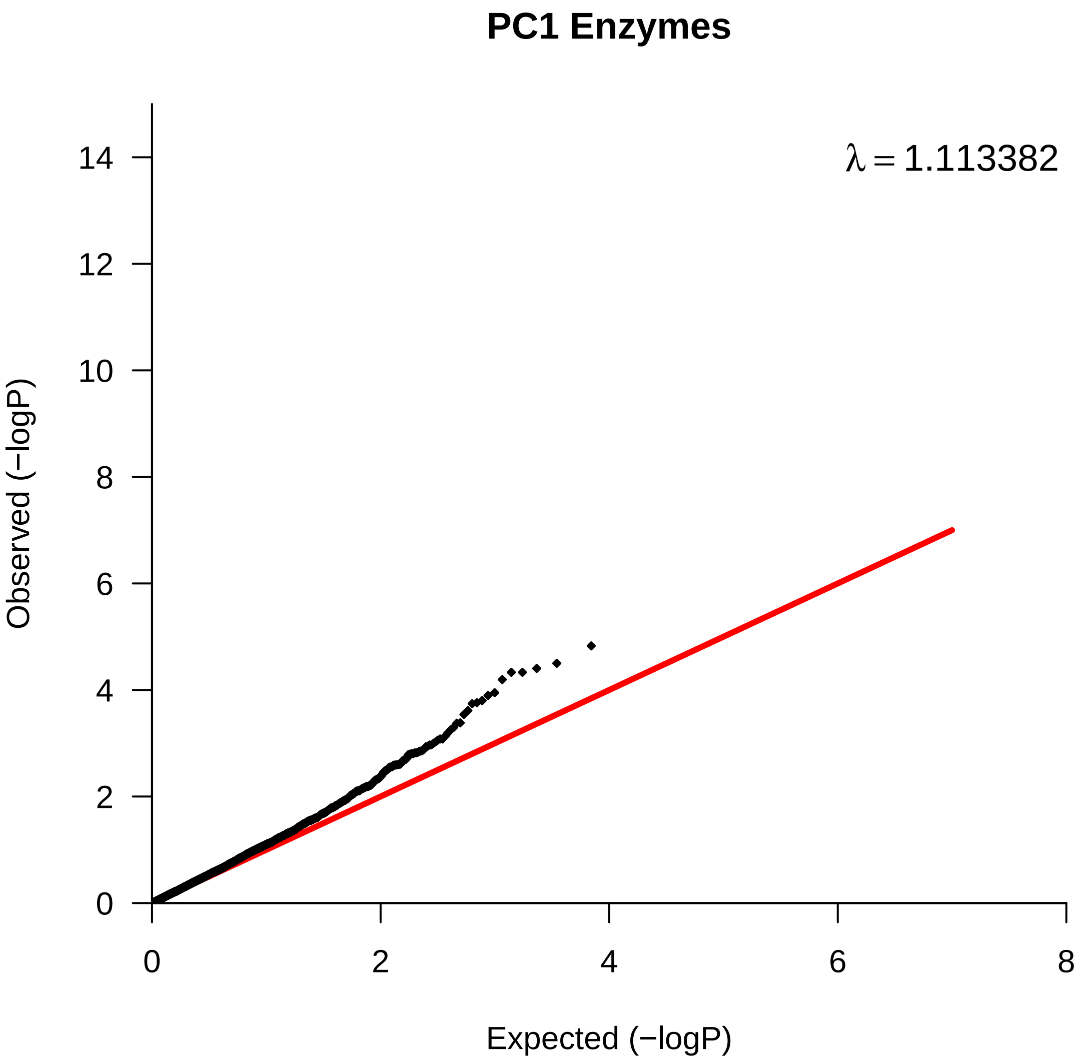

PC1 Enzymes
λ = 1.113382
Observed (−logP)
Expected (−logP)
0
2
4
6
8
10
12
14

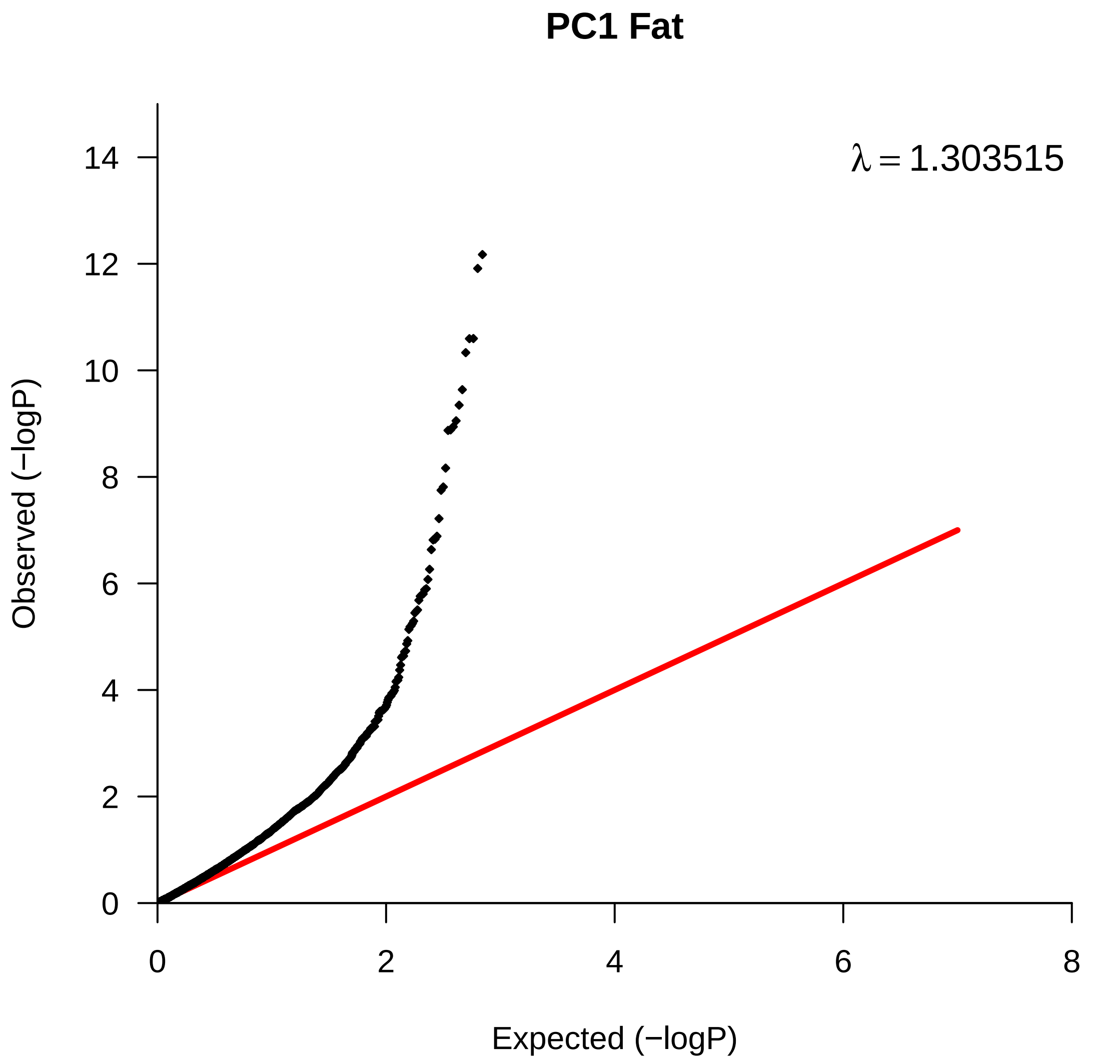
PC1 Fat
λ = 1.303515
Observed (−logP)
Expected (−logP)
0
2
4
6
8
10
12
14
0
2
4
6
8

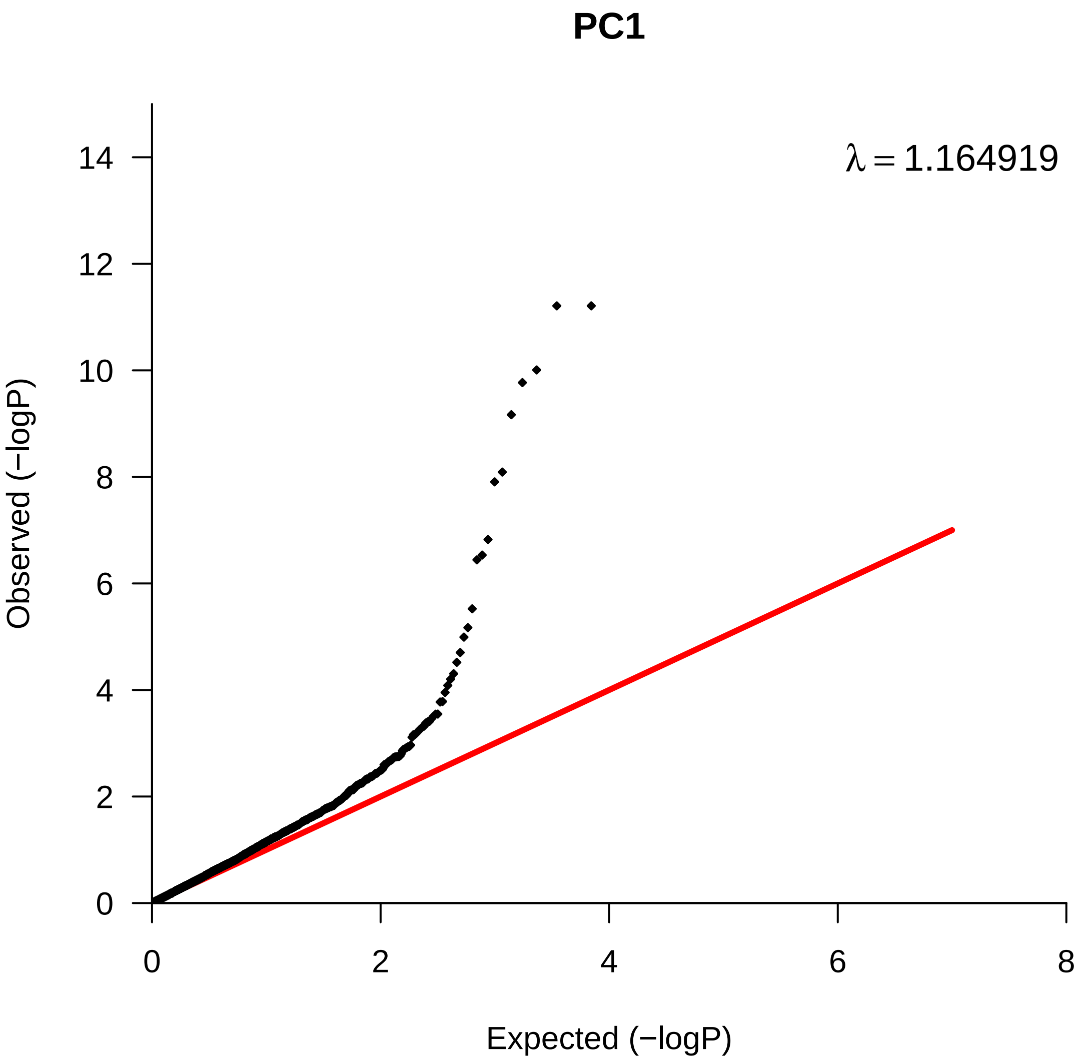
PC1
λ = 1.164919
Observed (−logP)
Expected (−logP)
0
2
4
6
8
10
12
14
0
2
4
6
8

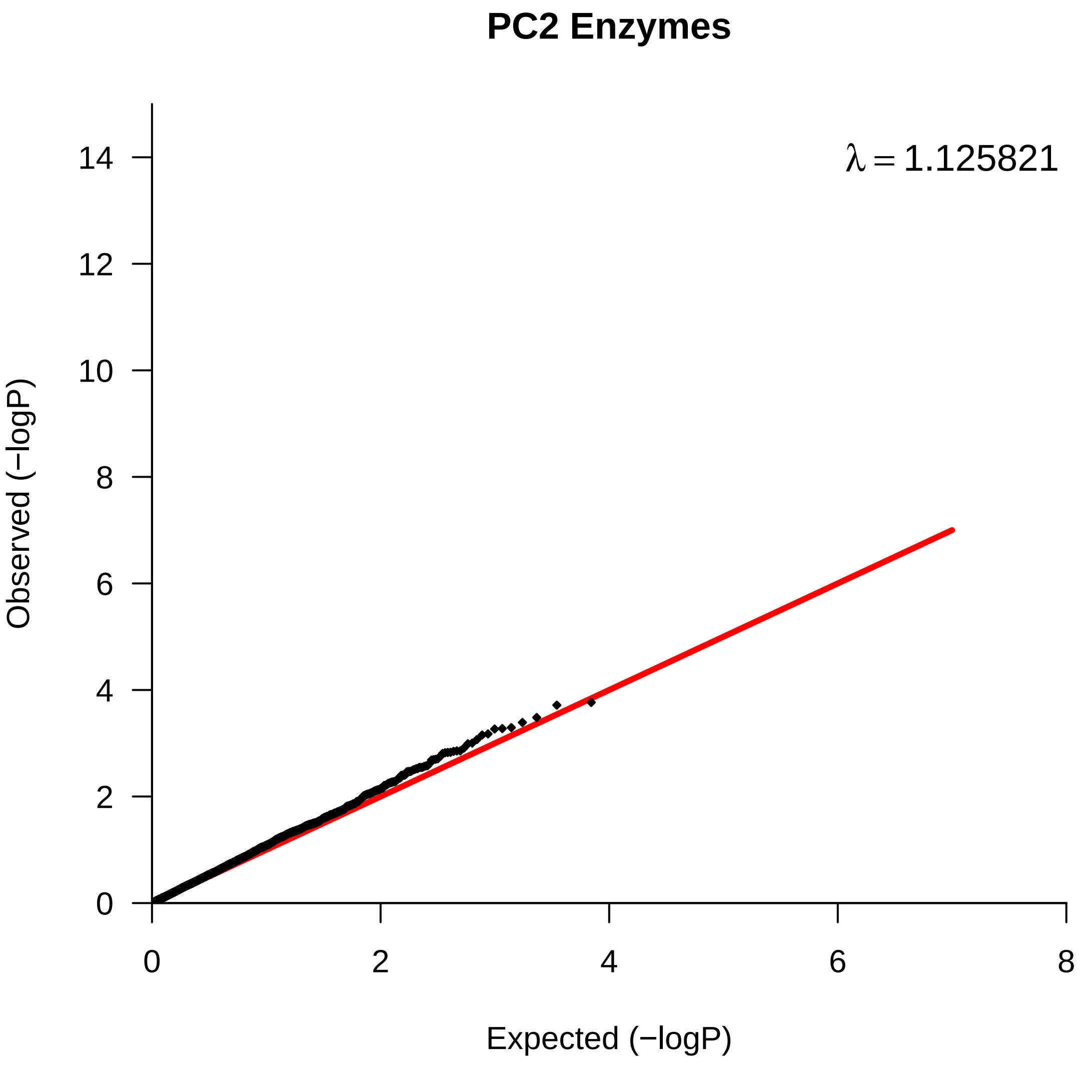
PC2 Enzymes
λ = 1.125821
Observed (−logP)
Expected (−logP)
0
2
4
6
8
10
12
14
0
2
4
6
8

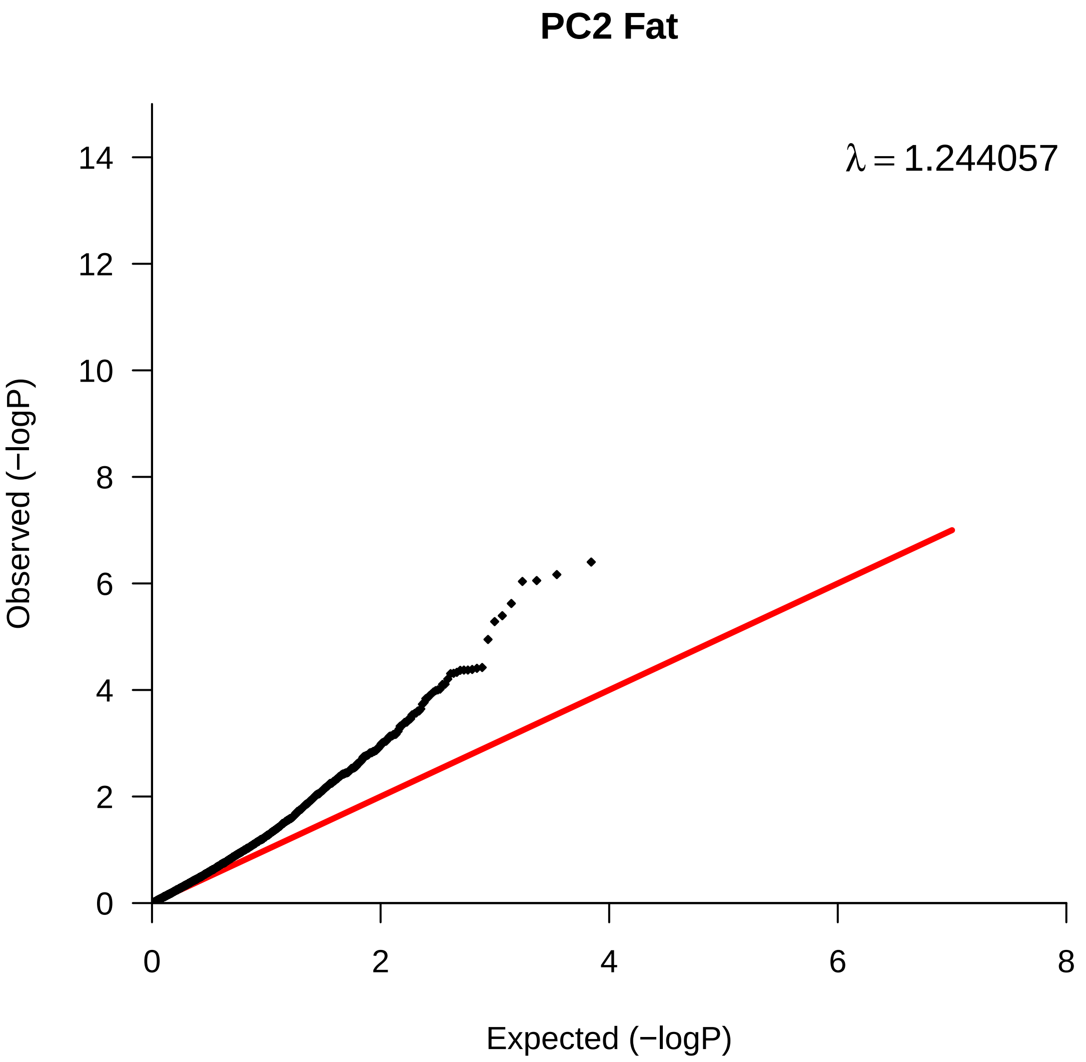
PC2 Fat
λ = 1.244057
Observed (−logP)
Expected (−logP)
0
2
4
6
8
10
12
14
0
2
4
6
8

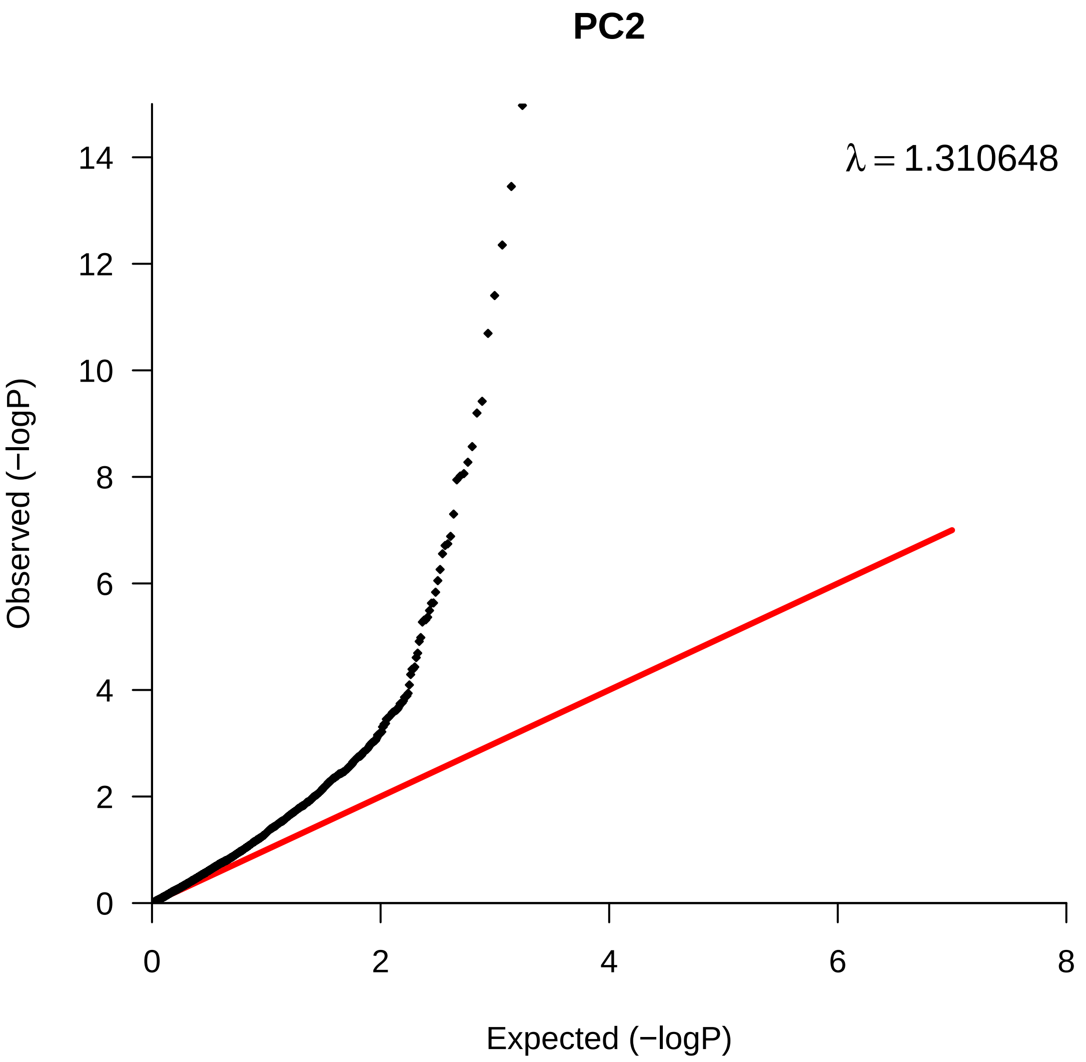
PC2
λ = 1.310648
Observed (−logP)
Expected (−logP)
0
2
4
6
8
10
12
14
0
2
4
6
8

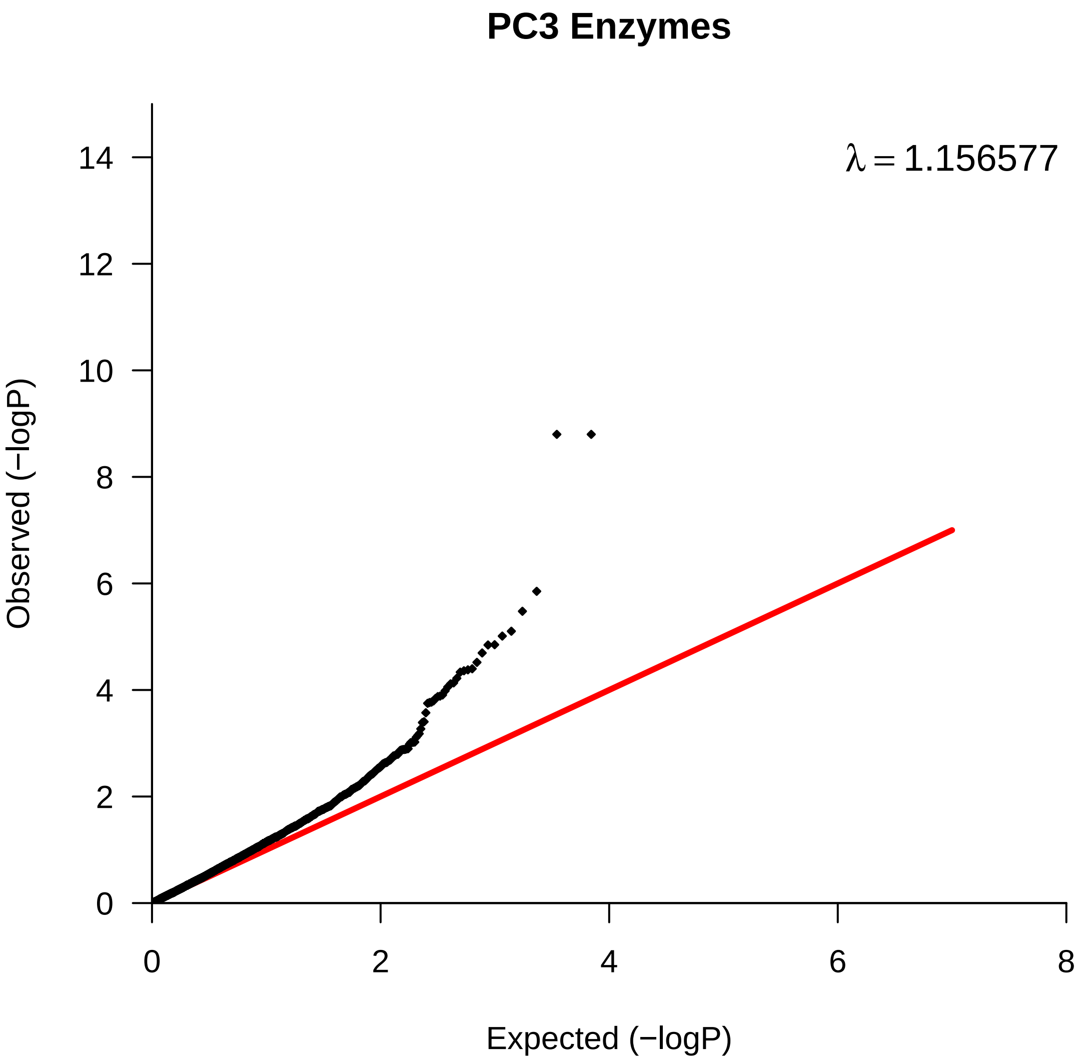
PC3 Enzymes
λ = 1.156577
Observed (−logP)
Expected (−logP)
0
2
4
6
8
10
12
14
0
2
4
6
8

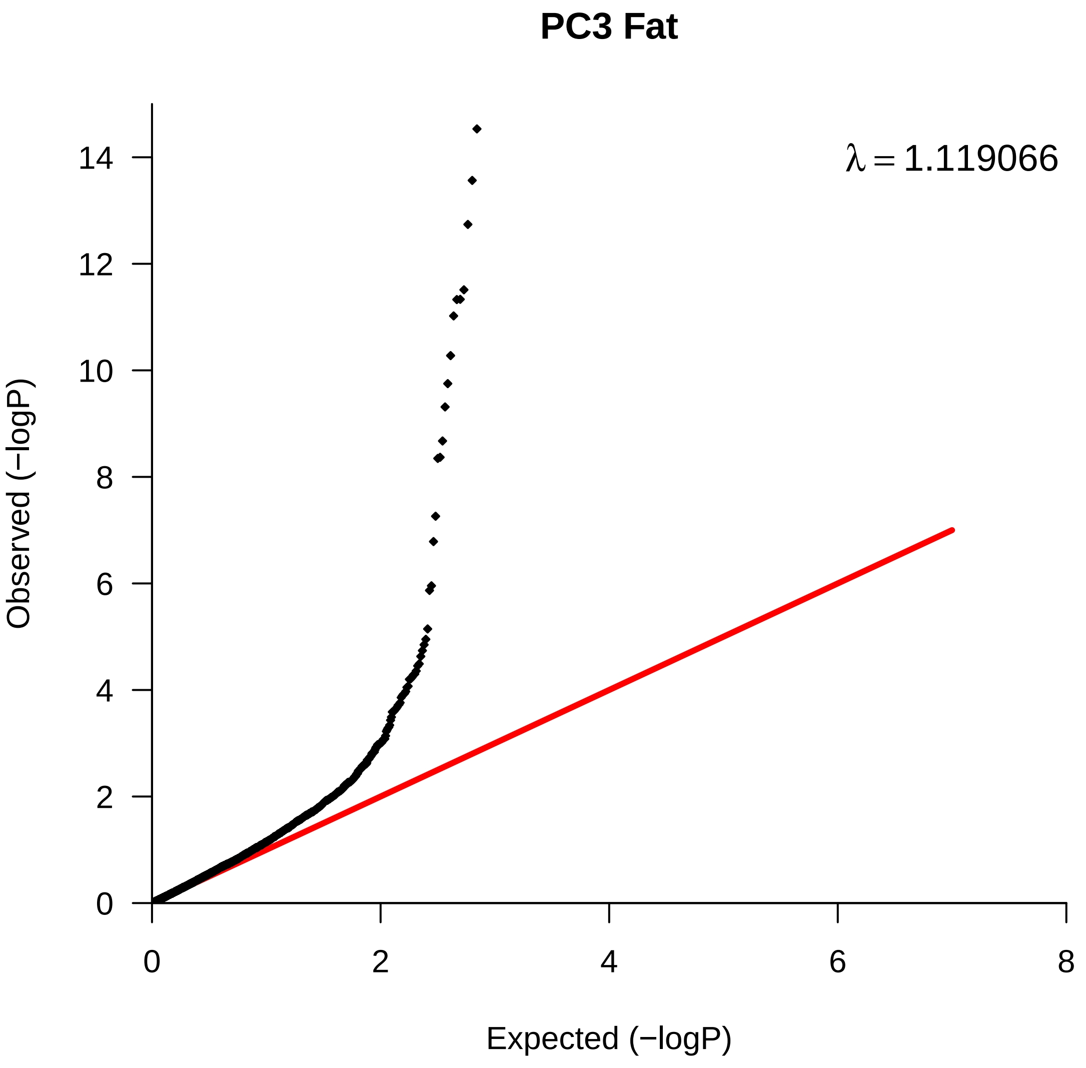
PC3 Fat
λ = 1.119066
Observed (−logP)
Expected (−logP)
0
2
4
6
8
10
12
14
0
2
4
6
8

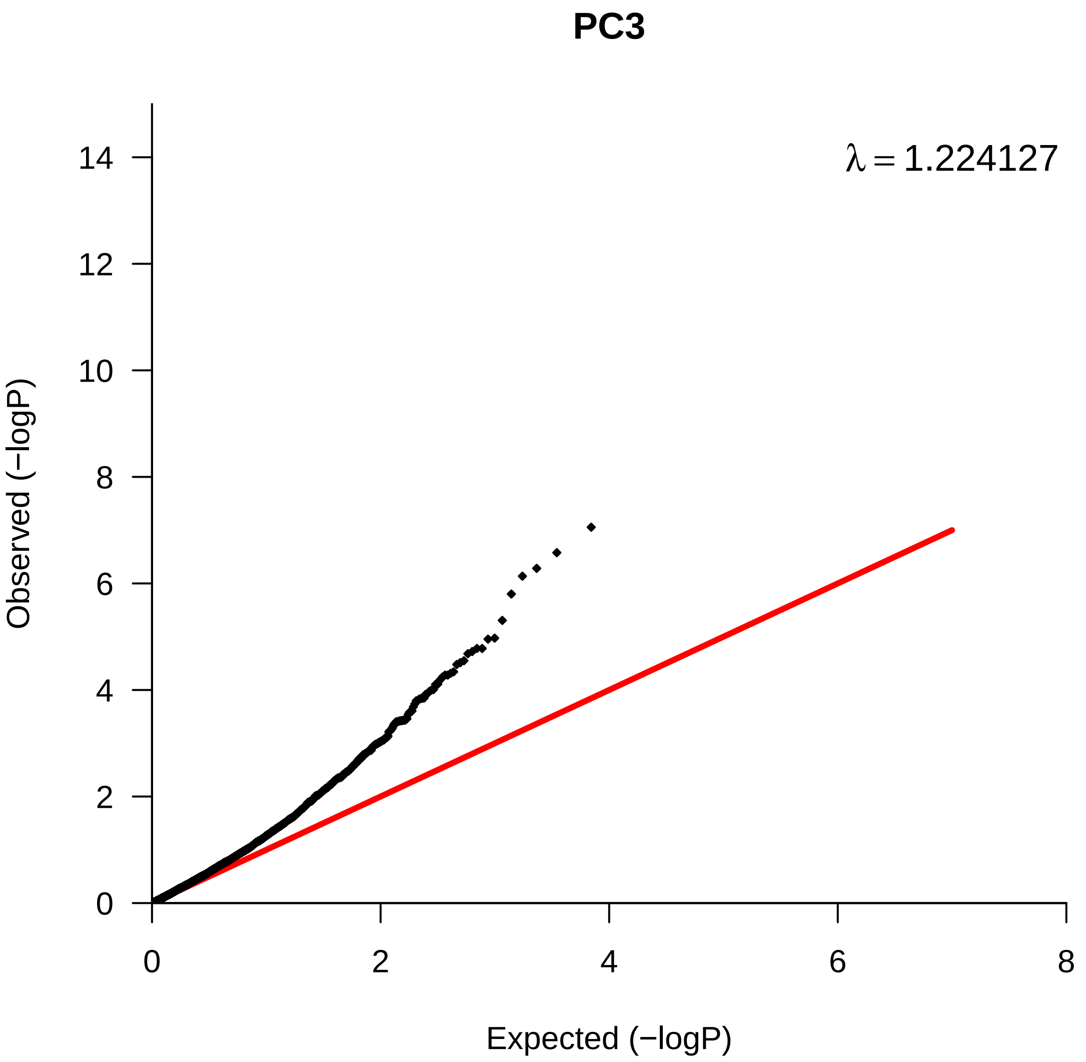
PC3
λ = 1.224127
Observed (−logP)
Expected (−logP)
0
2
4
6
8
10
12
14
0
2
4
6
8

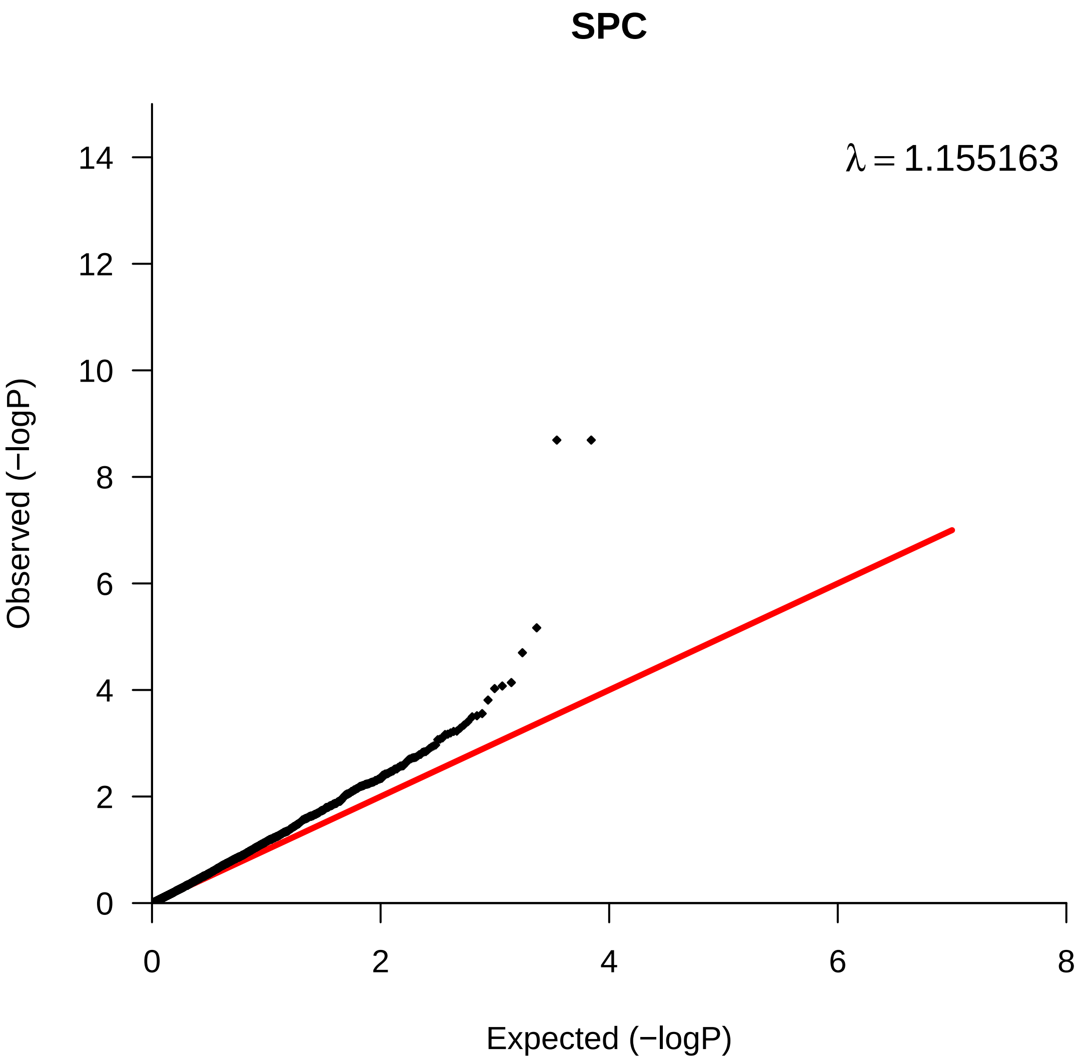
SPC
λ = 1.155163
Observed (−logP)
Expected (−logP)
0
2
4
6
8
10
12
14
0
2
4
6
8
