# Supplementary figures and images for: Large deletions in the DNA primase large subunit PRIM2 are associated with NADP‐malate dehydrogenase activity in a porcine F2 cross
Source: Anim Genet. 2026 Feb 2;57(1):e70077. doi: 10.1002/age.70077 (PMC12864183; doi:10.1002/age.70077)

PC\_AII

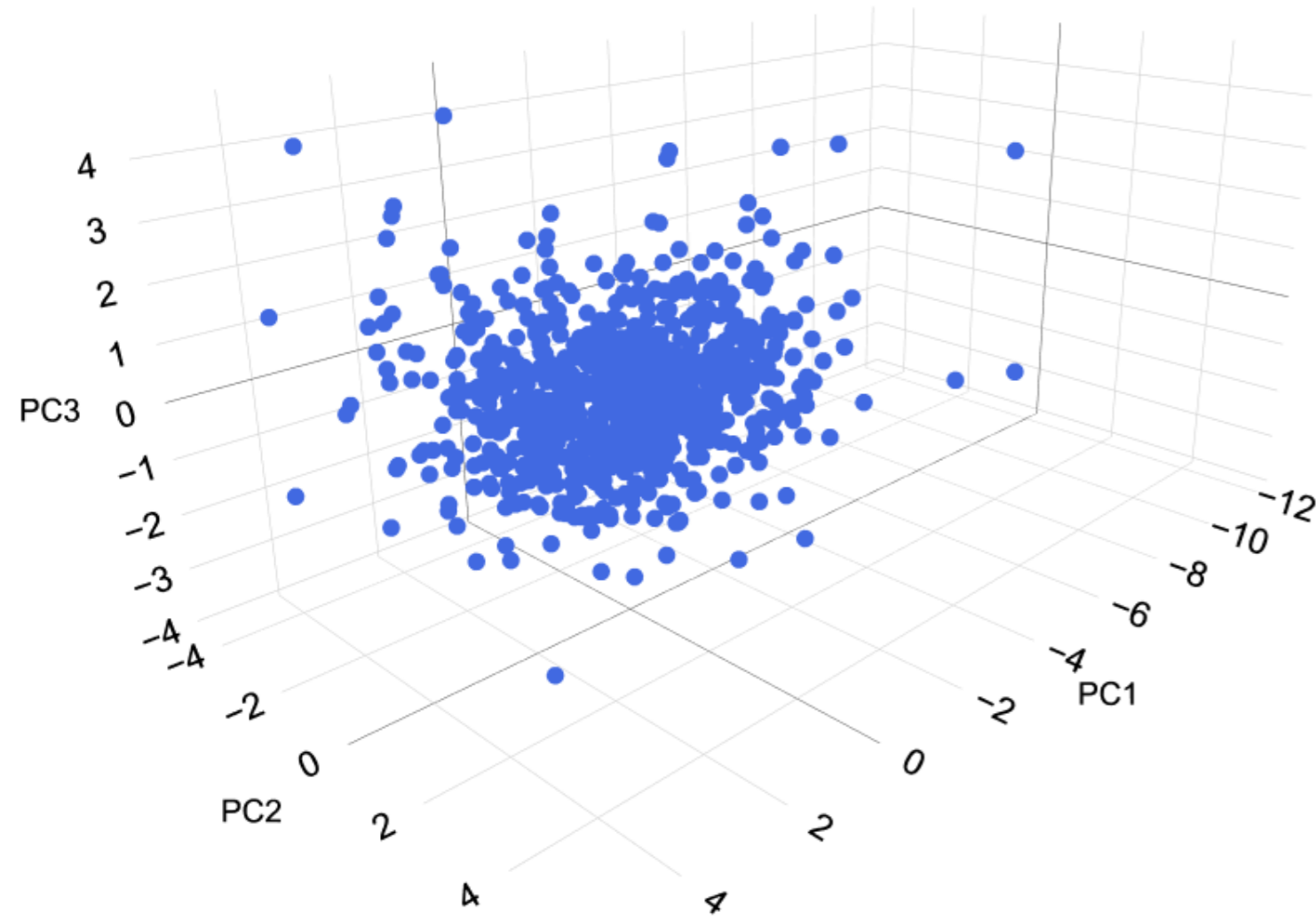

PC\_Enzymes

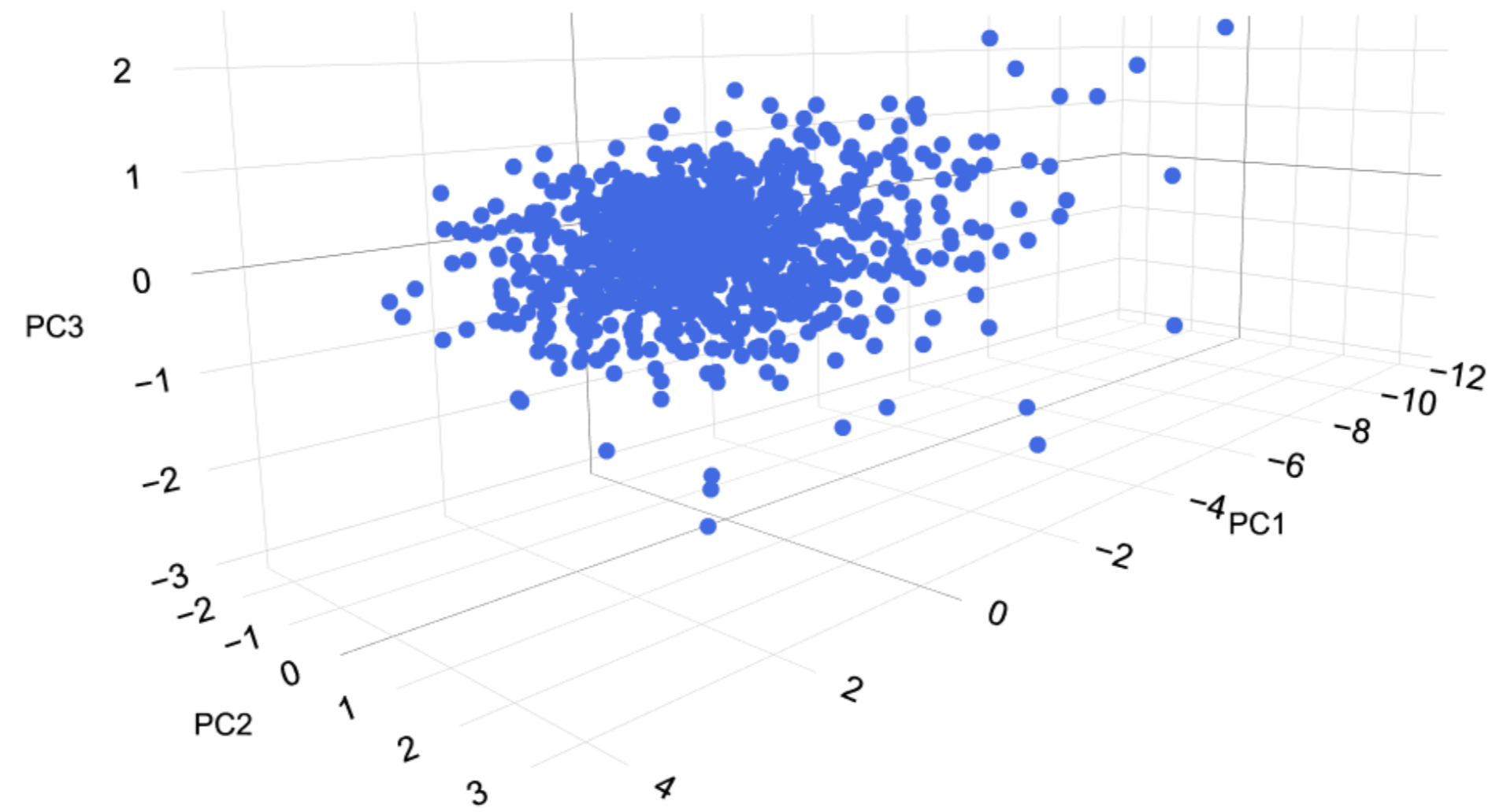

PC\_Fat

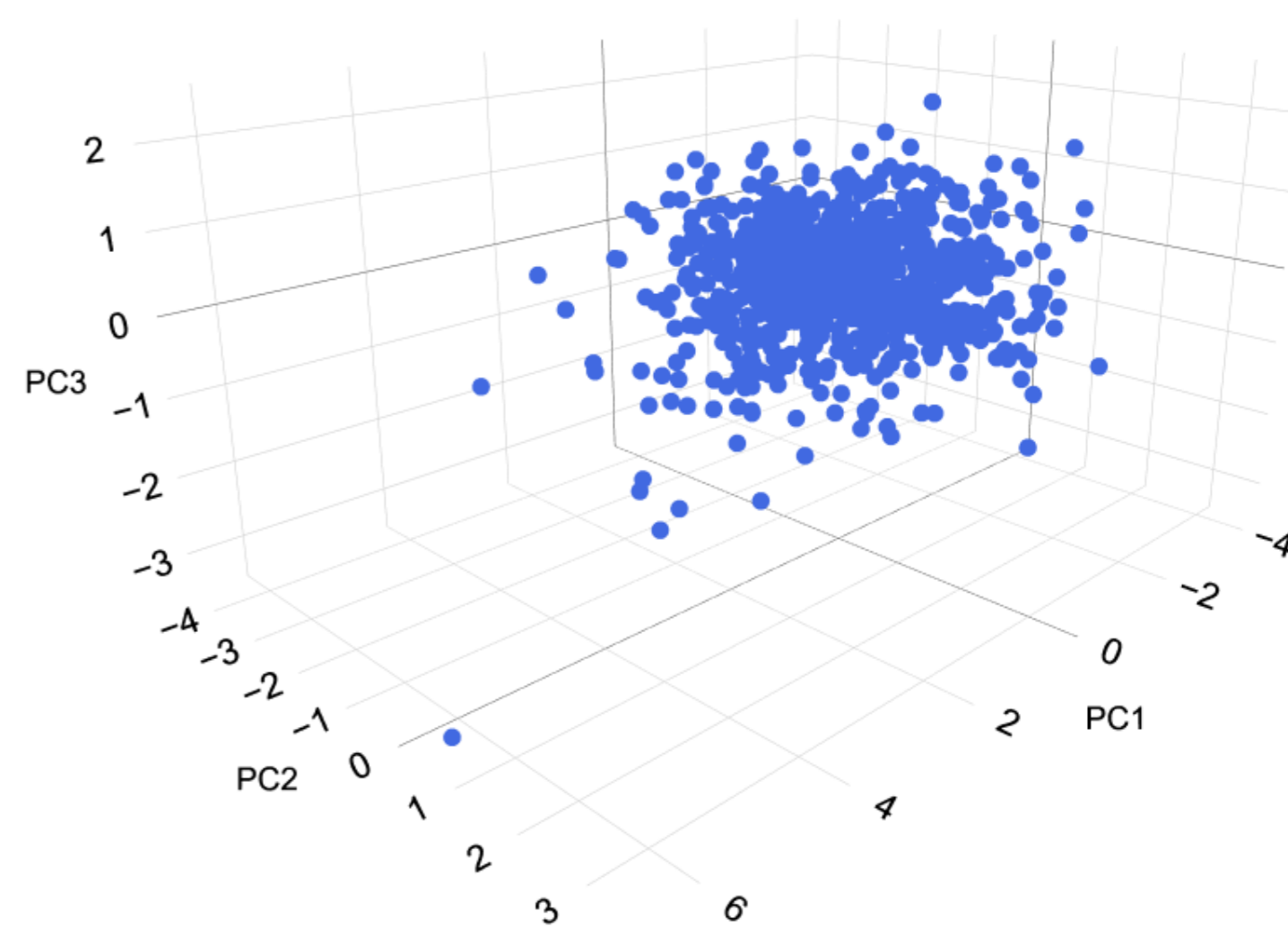

Supplement: Supplementary file 3 — Fig. S1: [file AGE-57-0-s005.pdf]

6PGD

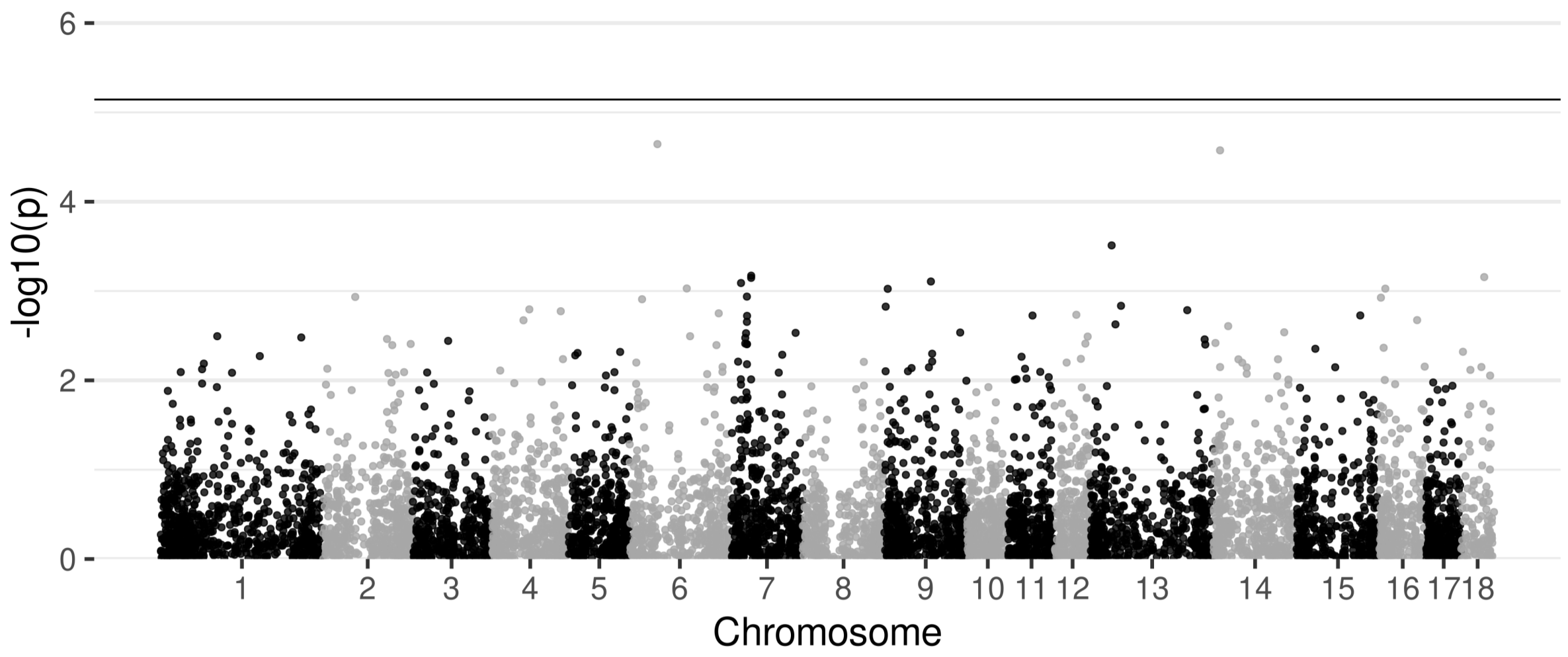

G6PD

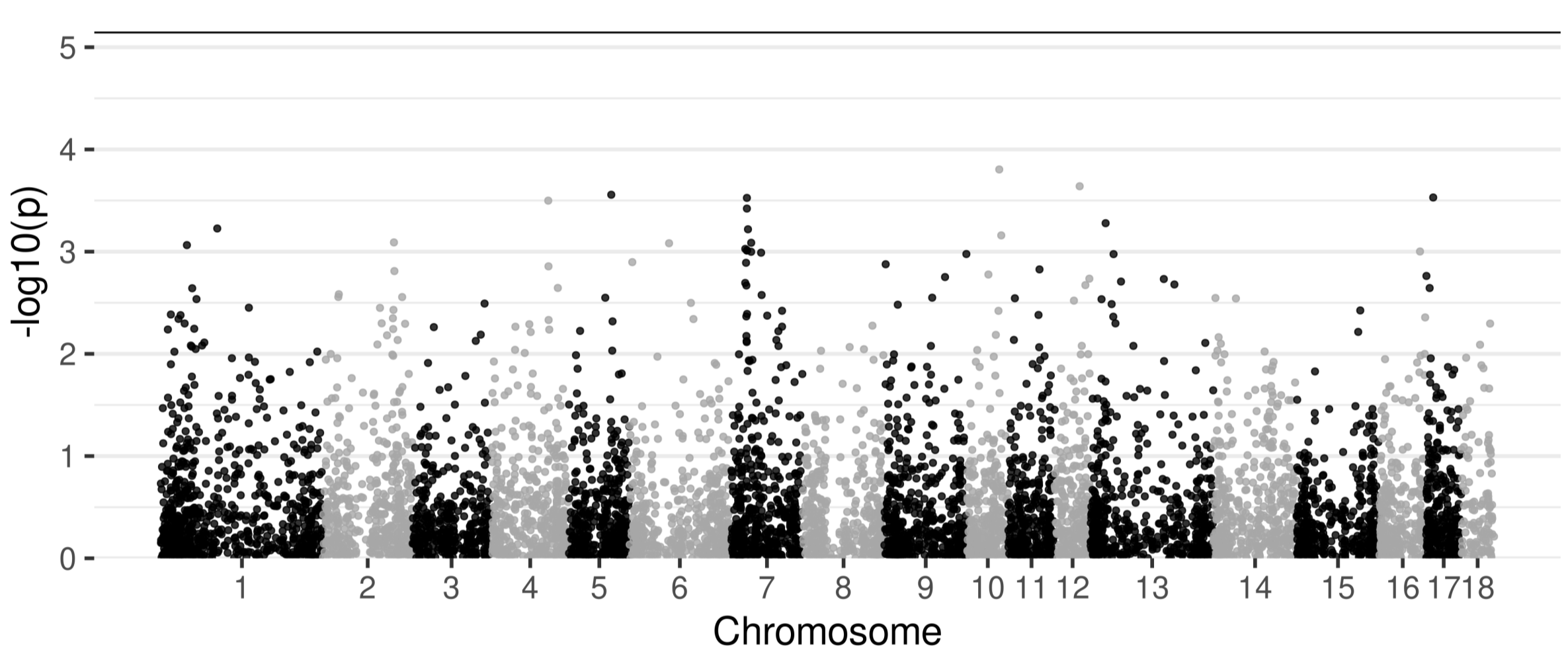

IDH

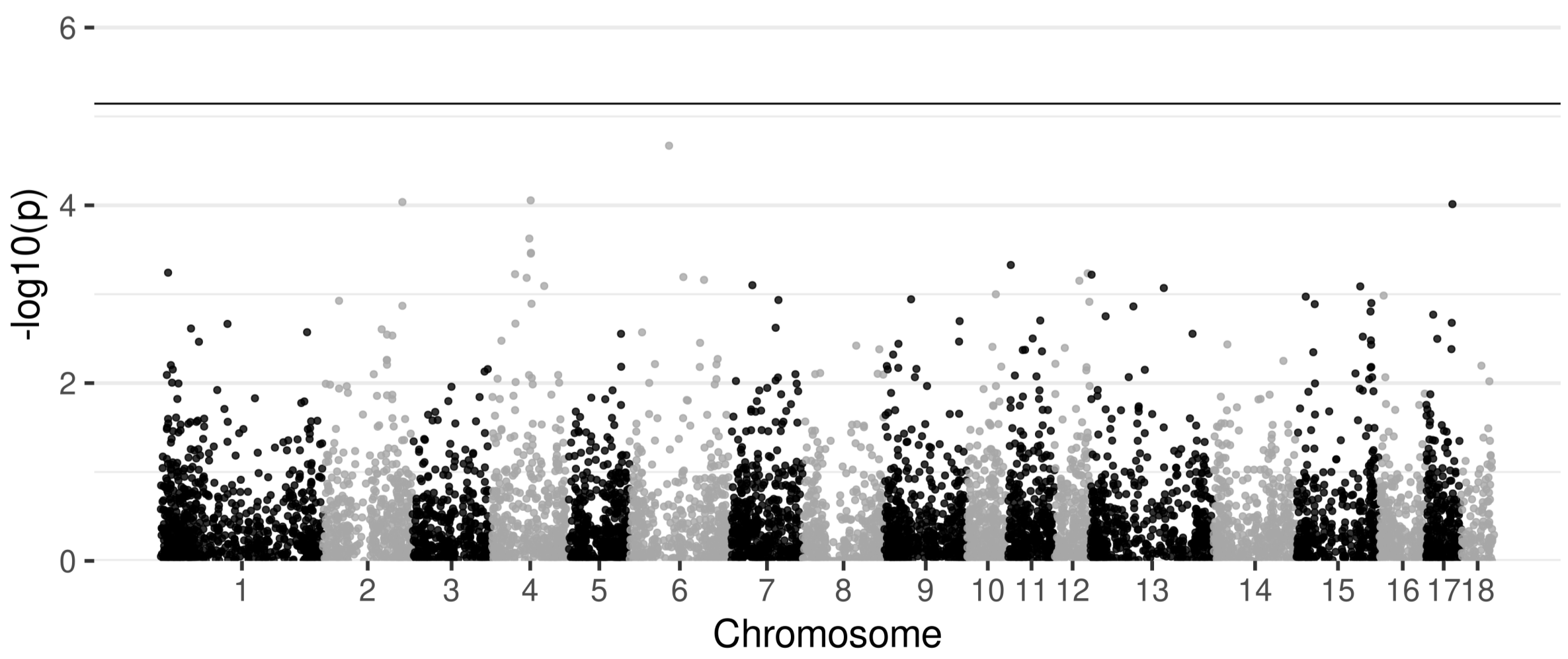

Supplement: Supplementary file 4 — Fig. S2: [file AGE-57-0-s007.pdf]

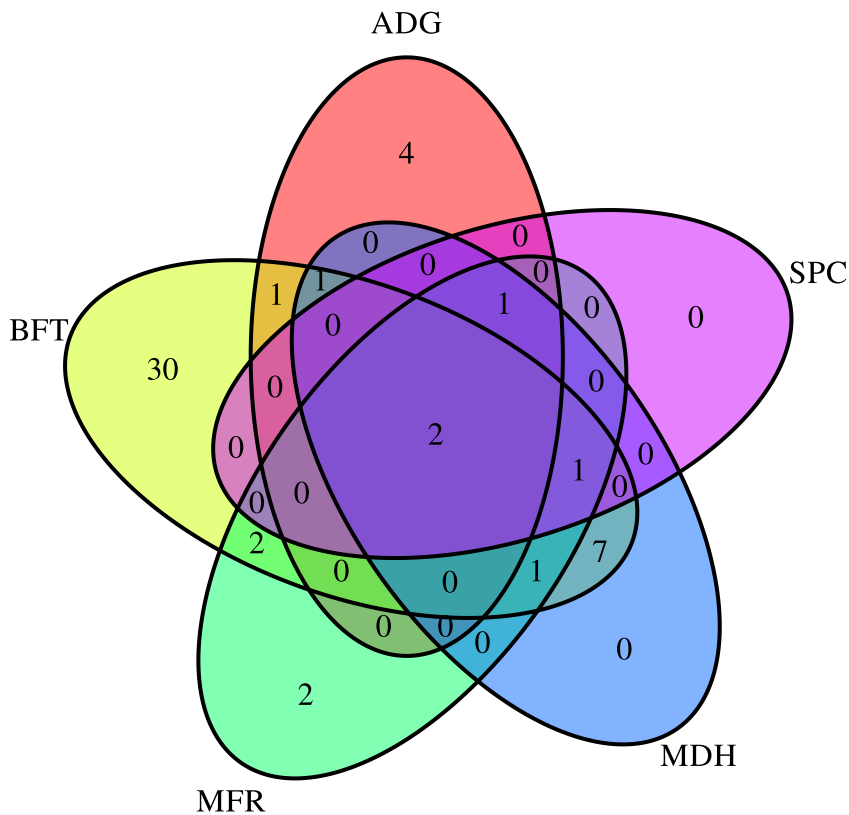

Supplement: Supplementary file 5 — Fig. S3: [file AGE-57-0-s003.pdf]

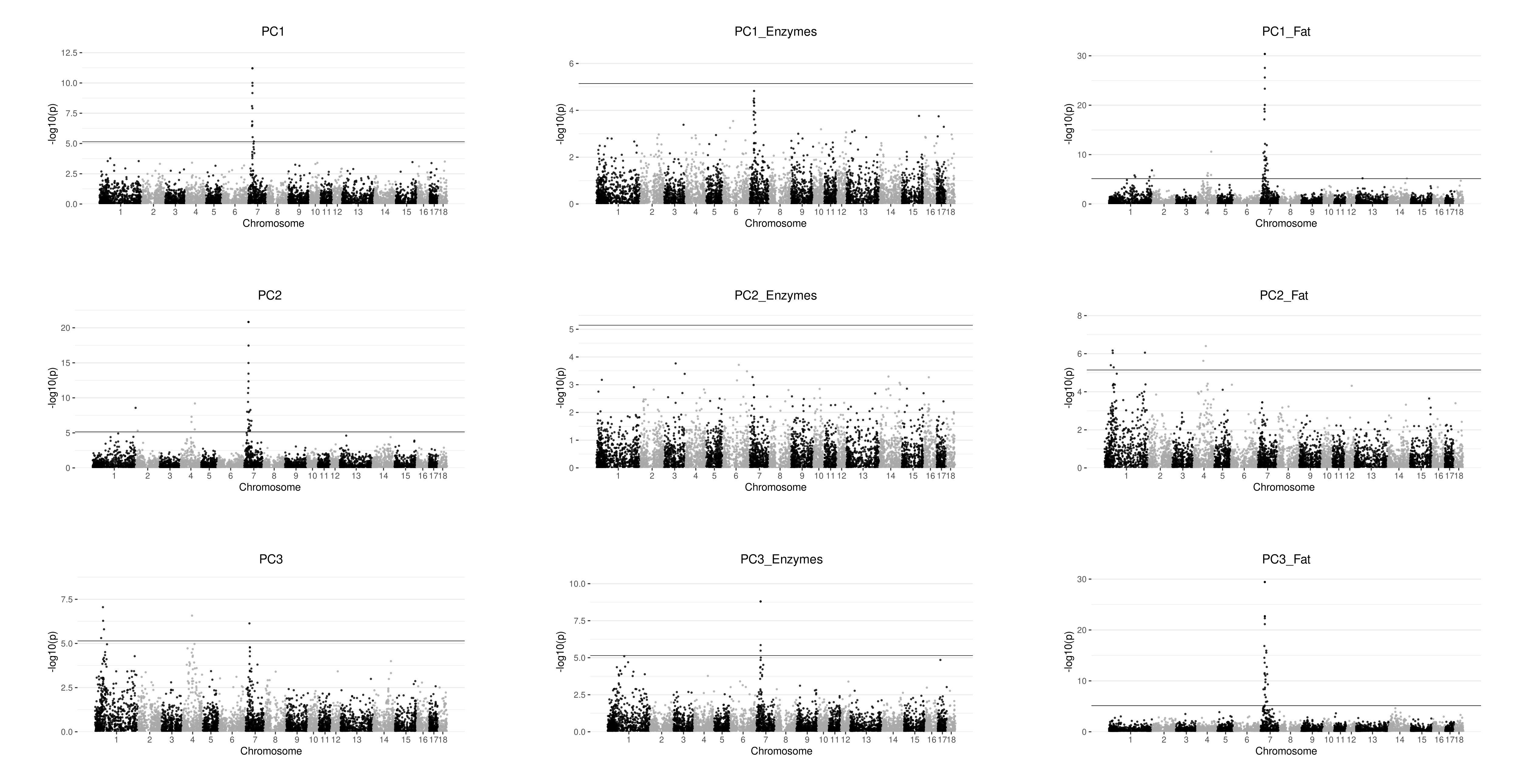

Supplement: Supplementary file 6 — Fig. S4: [file AGE-57-0-s008.png]

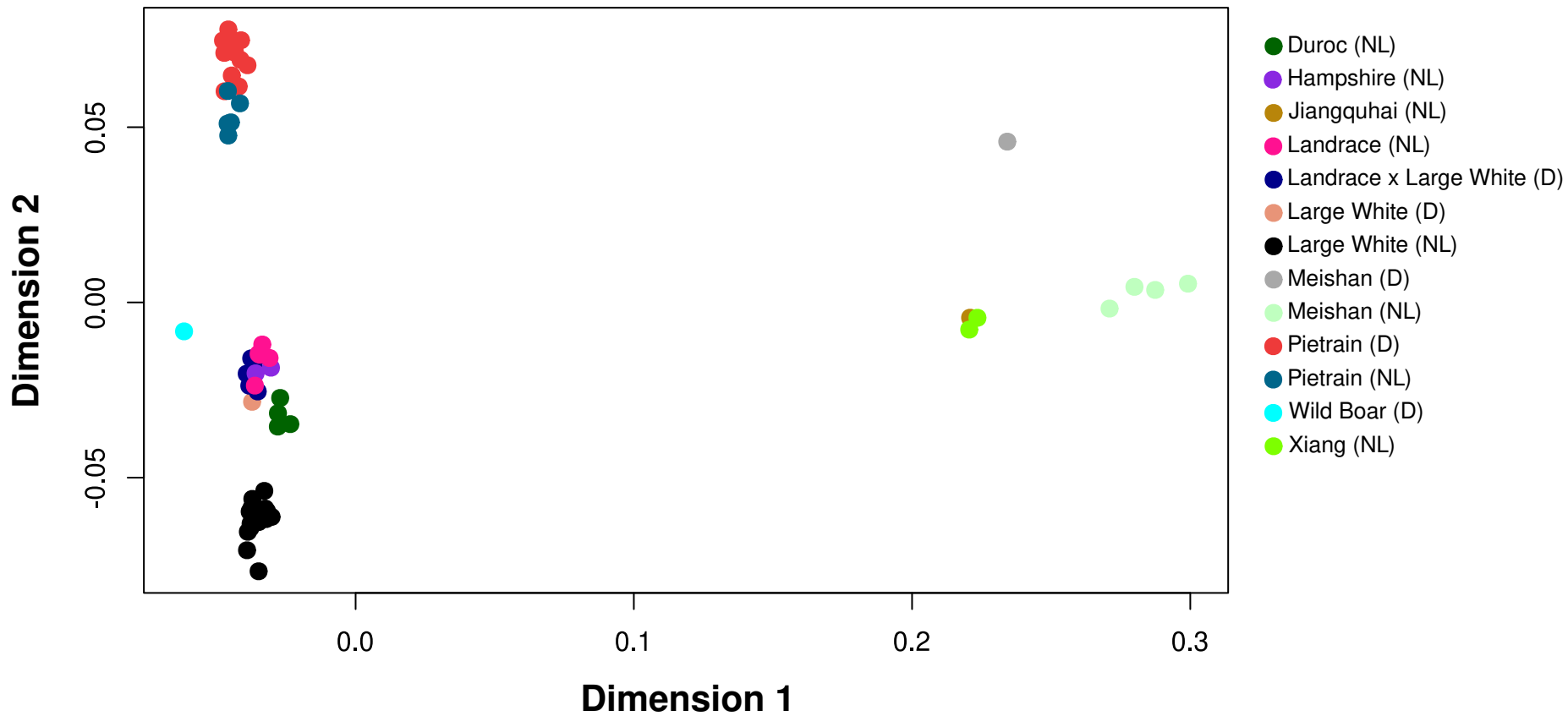

Supplement: Supplementary file 7 — Fig. S5: [file AGE-57-0-s001.pdf]
