# Supplementary material for: Large deletions in the DNA primase large subunit PRIM2 are associated with NADP‐malate dehydrogenase activity in a porcine F2 cross
Source: Anim Genet. 2026 Feb 2;57(1):e70077. doi: 10.1002/age.70077 (PMC12864183; doi:10.1002/age.70077)
Supplement: Supplementary file 8 — Table S1: [file AGE-57-0-s004.docx]

# SUPPLEMENTAL TABLES

Table S1: Proportion of variance explained by the first three principal components (PCs) obtained from all traits (all), only fat and muscle-related traits (fat), and only muscle enzyme traits (enzyme).

| **Traits** | **PC1** | **PC2** | **PC3** |
| --- | --- | --- | --- |
| all | 40.17% | 19.30% | 13.42% |
| fat | 52.45% | 33.20% | 14.35% |
| enzyme | 68.99% | 12.00% | 10.68% |
